# Functional analysis of candidate genes from genome-wide association studies of hearing

**DOI:** 10.1016/j.heares.2019.107879

**Published:** 2020-03-01

**Authors:** Neil J. Ingham, Victoria Rook, Francesca Di Domenico, Elysia James, Morag A. Lewis, Giorgia Girotto, Annalisa Buniello, Karen P. Steel

**Affiliations:** a Wolfson Centre for Age-Related Diseases, King’s College London, London, SE1 1UL, UK; b Wellcome Trust Sanger Institute, Hinxton, CB10 1SA, UK; c Clinical Department of Medical, Surgical and Health Sciences, University of Trieste, Institute for Maternal and Child Health, IRCCS Burlo Garofolo, Trieste, Italy

**Keywords:** Age-related hearing loss, Genome-wide association studies, Gene expression, Mammalian auditory system, Mouse mutants, Auditory brainstem response, Evoked potentials, *Dclk1*, *A430005L14Rik*, ABR, auditory brainstem response, ANOVA, analysis of variance, ARHL, age-related hearing loss, df, degrees of freedom, EP, endocochlear potential, GWAS, genome-wide association studies, qRT-PCR, quantitative real time polymerase chain reaction

## Abstract

The underlying causes of age-related hearing loss (ARHL) are not well understood, but it is clear from heritability estimates that genetics plays a role in addition to environmental factors. Genome-wide association studies (GWAS) in human populations can point to candidate genes that may be involved in ARHL, but follow-up analysis is needed to assess the role of these genes in the disease process. Some genetic variants may contribute a small amount to a disease, while other variants may have a large effect size, but the genetic architecture of ARHL is not yet well-defined. In this study, we asked if a set of 17 candidate genes highlighted by early GWAS reports of ARHL have detectable effects on hearing by knocking down expression levels of each gene in the mouse and analysing auditory function. We found two of the genes have an impact on hearing. Mutation of *Dclk1* led to late-onset progressive increase in ABR thresholds and the *A430005L14Rik* (*C1orf174*) mutants showed worse recovery from noise-induced damage than controls. We did not detect any abnormal responses in the remaining 15 mutant lines either in thresholds or from our battery of suprathreshold ABR tests, and we discuss the possible reasons for this.

## Highlights

• *Dclk1* may play a role in progressive age-related hearing loss.
• *A430005L14Rik* may play a role in the susceptibility of the auditory system to noise-damage.
• Fifteen other candidate genes (from GWAS) knocked out or knocked down in mutant mice do not lead to changes in ABRs.

## 1 Introduction

Age-related progressive hearing loss (ARHL) is very common in the population and can begin at any age. One in 850 children is born with hearing impairment and with each decade this number increases until over half of adults in their 70s have a significant hearing loss (Fortnum et al., 2001; Davis, 1995). Hearing impairment is profoundly isolating, both socially and economically, and has a major impact on the quality of life of those affected, often associated with depression or cognitive decline, and is a predictor of dementia (Fellinger et al., 2012; Karpa et al., 2010; Mick et al., 2014; Livingston et al., 2017). People often first show difficulty in following conversations in a noisy background, followed by progressive loss of auditory sensitivity starting with high tones. However, thresholds for detecting sound do not always reflect the degree of dysfunction experienced by people in the early stages of progressive hearing loss. The only remedial options currently available for ARHL are hearing aids and cochlear implants, which may provide some benefits but do not restore normal function. There is a large unmet need for medical approaches to slow down or reverse progressive hearing loss. Developing treatments will require knowledge of the molecular and cellular basis of ARHL, and genetics is a useful tool to give us insight into the molecular networks and pathways involved.

ARHL is a heterogeneous disorder. Environmental factors such as noise, drugs and infections contribute to it, but little is known about the genetic factors involved. A number of single gene mutations leading to progressive hearing loss with onset in adulthood have been identified, mostly showing dominant inheritance, however few of these affect more than a handful of families (see Van Camp and Smith, 2019). Twin, sib, and family studies of hearing ability have revealed heritabilities up to 0.7 or greater, indicating a significant contribution of genetics (Karlsson et al., 1997; Gates et al., 1999; Wolber et al., 2012; Viljanen et al., 2007; Demeester et al., 2010). One approach to finding genetic variants that are common in the population and are associated with a phenotype is the Genome Wide Association Study (GWAS), which screens large numbers of variable genetic markers distributed widely around the genome and looks for association between variants and the trait of interest. Although several ARHL GWAS have been carried out (eg Girotto et al., 2011, 2014; Huyghe et al., 2008; Friedman et al., 2009; Nolan et al., 2013; Wolber et al., 2014; Fransen et al., 2015) and promising candidate genes identified, such as *SIK3* and *ESRRG*, until recently only five loci were associated with hearing status at the genome-wide significance level: *GRM7* (Friedman et al., 2009), *PCDH20* and *SLC28A3* (Vuckovic et al., 2015), and *ISG20* or *ACAN* and *TRIOBP* (Hoffman et al., 2016). At least one of these candidate genes (*TRIOBP*) is also known to underlie childhood deafness, where single gene mutations segregate with the deafness (Riazuddin et al., 2006; Shahin et al., 2006), suggesting that other genes known to underlie childhood deafness may also be candidates for ARHL and *vice versa*. Further support for the suggestion that there is an overlap between genes involved in childhood deafness and those associated with ARHL has come from two very recent independent GWAS analyses of the UK Biobank data. Wells et al. (2019) reported 10 of the 44 associated loci included genes known to be involved in Mendelian deafness, and Kalra et al. (2019) found a similar proportion among the 50 significant loci they listed. However, neither study replicated the previous associations of hearing status with *GRM7*, *PCDH20*, or *SLC28A3*.

Common diseases with adult onset, like ARHL, are often considered to be associated with many genetic variants each having a small effect size. However, the finding of a number of single-gene variants with a large effect size and Mendelian inheritance causing progressive hearing loss (39 out of 44 dominantly-inherited nonsyndromic genes involved in deafness, Van Camp and Smith, 2019) suggests that there may be further single-gene mutations predisposing to hearing loss contributing to ARHL in the population. The genetic architecture of ARHL remains to be defined, but any clues to the molecular pathways underlying hearing loss from single-gene mutations will be valuable in understanding the molecular pathophysiology and hence facilitate the development of treatments for the condition.

In this study, we follow up some of the candidate genes from early GWAS reports (Girotto et al., 2011, 2014; Wolber et al., 2014) by detailed testing of auditory function in mouse mutants with the orthologous genes knocked-out or knocked-down. It is unlikely that the candidate genes are inactivated completely in humans with ARHL, but we can use severe (knockout) alleles in the mouse to identify and characterise any hearing loss in this extreme condition. Furthermore, as increased thresholds for detection of sounds is often preceded by other auditory difficulties in humans, we analysed the mouse mutants for more subtle defects in auditory function including frequency tuning and temporal processing measures which might serve as early indicators of dysfunction. We analysed a total of 17 genes and found one, *Dclk1*, that was associated with late-onset hearing loss and one, *A430005L14Rik*, that affected auditory function following noise-induced damage.

## 2 Material and methods

### 2.1 Selection of genes

The genes selected are listed in Table 1 and were identified as potentially involved in ARHL in three reports of GWAS carried out on populations in Southern Europe, the Silk Road and the UK (Girotto et al., 2011, 2014; Wolber et al., 2014). These studies took a meta-analytic approach, combining data from multiple populations to obtain a total number of subjects of 3417 (Girotto et al., 2011) and 4939 (Wolber et al., 2014). For each peak GWAS variant, we investigated genes located within 250 kb up- or downstream of the marker and filtered them based on expression in the cochlea and potential functional links to genes known to be involved in hearing loss (for a full description of the selection criteria, see Girotto et al., 2014). Previous immunolabelling studies of expression in the mouse revealed distinctive cochlear expression patterns in several of these genes (Girotto et al., 2014). Three showed expression restricted to hair cells (*Csmd1, Arsg, Slc16a6*), one was expressed only in marginal cells of the stria vascularis (*Dclk1*) and others (*Ptprd, Grm8, Evi5, Rimbp2, Sik3*) showed expression in multiple cochlear cell types (Table 2).

**Table 1:** **Genes tested**.

Gene	Gene name	Human orthologue	Source of candidate	Peak variant marker	p value of association	Variant to gene distance or variant position within gene	DNA Strand
*A430005L14Rik*	RIKEN cDNA A430005L14 gene	*C1orf174*	Girotto et al. (2011)	rs6673959	7.97E-06	∼163 kb	+
*Amz2*	Archaelysin family metallopeptidase 2	*AMZ2*	Girotto et al. (2014)	rs8077384	9.66E-06	∼39 kb	+
*Arsg*	Arylsulfatase G	*ARSG*	Girotto et al. (2014)	rs8077384	9.66E-06	∼93 kb	+
*Dclk1*	Doublecortin-like kinase 1	*DCLK1*	Girotto et al. (2014)	rs9574464	3.18E-07	∼30 kb	+
*Evi5*	Ecotropic viral integration site 5	*EVI5*	Girotto et al. (2014)	rs12758887	8.68E-06	∼136 kb	–
*Fzd6*	Frizzled class receptor 6	*FZD6*	Girotto et al. (2011)	rs1110115	9.54E-06	∼212 kb	+
*Grm8*	Glutamate receptor, metabotropic 8	*GRM8*	Girotto et al. (2014)	rs2687481	3.22E-07	∼210 kb	–
*Ptprd*	Protein tyrosine phosphatase, receptor type, D	*PTPRD*	Girotto et al. (2014)	rs10815873	3.35E-07	NM_002839.3:c.4086 + 398C.A	–
*Sik3*	SIK family kinase3	*SIK3*	Wolber et al. (2014)	rs681524	3.69E-08	NM_025164.6:c.866-556A > G	+
*Slc16a6*	Solute carrier family 16, member 6	*SLC16A6*	Girotto et al. (2014)	rs8077384	9.66E-06	∼100 kb	–
*Tgm6*	Transglutaminase 6	*TGM6*	Unpublished (Girotto, G.)	rs2422764	<1.0E-05	NM_198994.3:c.7 + 6447C > G	+
*Cmip*	c-Maf inducing protein	*CMIP*	Girotto et al. (2011)	rs1563655	8.61E-06	∼3 kb	+
*Csmd1*	CUB and Sushi multiple domains 1	*CSMD1*	Girotto et al. (2014)	rs10091102	8.75E-05	NM_033225.5:c.498521219C.G	–
*Dipk1a (Fam69a)*	Divergent protein kinase domain 1A	*DIPK1A*	Unpublished (Girotto, G.)	rs12758887	8.68E-06	NM_001006605.5:c.54 + 32878C > T	–
*Optn*	Optineurin	*OPTN*	Girotto et al. (2011)	rs641113	3.66E-06	∼4 kb	–
*Pthlh*	parathyroid hormone-like peptide	*PTHLH*	Girotto et al. (2011)	Gene based on network analysis			–
*Rimbp2*	RIMS binding protein 2	*RIMBP2*	Girotto et al. (2014)	rs10848114	4.75E-06	NM_015347.4:c.24 + 11838G.A	–

**Table 2:** **Genes tested, Mutations, Gene expression patterns, Physiological tests performed, and other known phenotypes.** Mutation Key: T-CR, Targeted, conditional-ready; T-NC, Targeted, non-conditional; EM-Exdel, Endonuclease-mediated Exon Deletion. Expression Key: CD, multiple cell types around cochlear duct; SV, stria vascularis; SG, spiral ganglion cells; HC, hair cells; OHC, outer hair cells; RM, Reissners’ membrane; (ND), not detected; –, not tested.

Gene	Allele	Mutation	Protein expression (Girotto et al. 2014)	LacZ reporter expression	Basic ABR	Frequency tuning and temporal processing	Noise exposure	Other mouse phenotypes (from IMPC, https://www.mousephenotype.org/)
*A430005L14Rik*	*A430005L14Rik*^*tm1a(KOMP)Wtsi*^	*T-CR*	–	SG, CD	Y	Y	Y	Decreased circulating insulin level
*Amz2*	*Amz2*^*tm1e(KOMP)Wtsi*^	*T-NC*	–	SG	Y	Y	–	Increased mature B cell number
*Arsg*	*Arsg*^*tm1a(KOMP)Wtsi*^	*T-CR*	HCs	CD	Y	Y	–	Decreased B cell number; thrombocytosis
*Dclk1*	*Dclk1*^*tm1a(EUCOMM)Wtsi*^	*T-CR*	SV	SV	Y	Y	–	Decreased haematocrit, circulating triglyceride level, erythrocyte cell number
*Evi5*	*Evi5*^*tm1a(KOMP)Wtsi*^	T-CR	CD	(ND)	Y	Y	–	None significant
*Fzd6*	*Fzd6*^*tm2a(EUCOMM)Wtsi*^	*T-CR*	–	CD, SV, SG	Y	Y	–	None significant
*Grm8*	*Grm8*^*tm2a(KOMP)Wtsi*^	*T-CR*	CD, SG	(ND)	Y	Y	Y	None significant
*Ptprd*	*Ptprd*^*tm2a(KOMP)Wtsi*^	*T-CR*	CD, SG	–	Y	Y	–	Different allele shows many phenotypes
*Sik3*	*Sik3*^*tm1a(EUCOMM)Hmgu*^	*T-CR*	HCs, RM, SV, SG	CD	Y	Y	–	Multiple abnormalities (79 measures)
*Slc16a6*	*Slc16a6*^*tm1a(EUCOMM)Wtsi*^	*T-CR*	OHCs	CD	Y	Y	–	None significant
*Tgm6*	*Tgm6*^*tm1a(KOMP)Wtsi*^	*T-CR*	–	SG, SV	Y	Y	–	Decreased circulating cholesterol level
*Cmip*	*Cmip*^*tm1a(EUCOMM)Wtsi*^	*T-CR*	–	–	Y	–	–	Homozygous lethal
*Csmd1*	*Csmd1*^*em1(IMPC)Wtsi*^	*EM-Exdel*	HCs	–	Y	–	–	Bone mineral/skeletal defects, increased creatinine levels, impaired glucose tolerance
*Dipk1a (Fam69a)*	*Dipk1a*^*tm1a(EUCOMM)Wtsi*^	*T-CR*	–	CD, SG	Y	–	–	Increased circulating alkaline phosphatase level, decreased cholesterol level
*Optn*	*Optn*^*tm1a(EUCOMM)Wtsi*^	*T-CR*	–	–	Y	–	–	Decreased circulating LDL and HDL cholesterol levels
*Pthlh*	*Pthlh*^*tm1a(KOMP)Wtsi*^	*T-CR*	–	–	Y	–	–	Homozygous lethal, abnormal head morphology in hets
*Rimbp2*	*Rimbp2*^*em1(IMPC)Wtsi*^	*EM-Exdel*	CD, SG	–	Y	–	–	None significant

### 2.2 Ethics statement

Mouse studies were carried out in accordance with UK Home Office regulations and the UK Animals (Scientific Procedures) Act of 1986 (ASPA) under UK Home Office licences, and the study was approved by the King’s College London Ethical Review Committee. Mice were culled using methods approved under these licences to minimise any possibility of suffering.

### 2.3 Mice

**Fig. 1:**
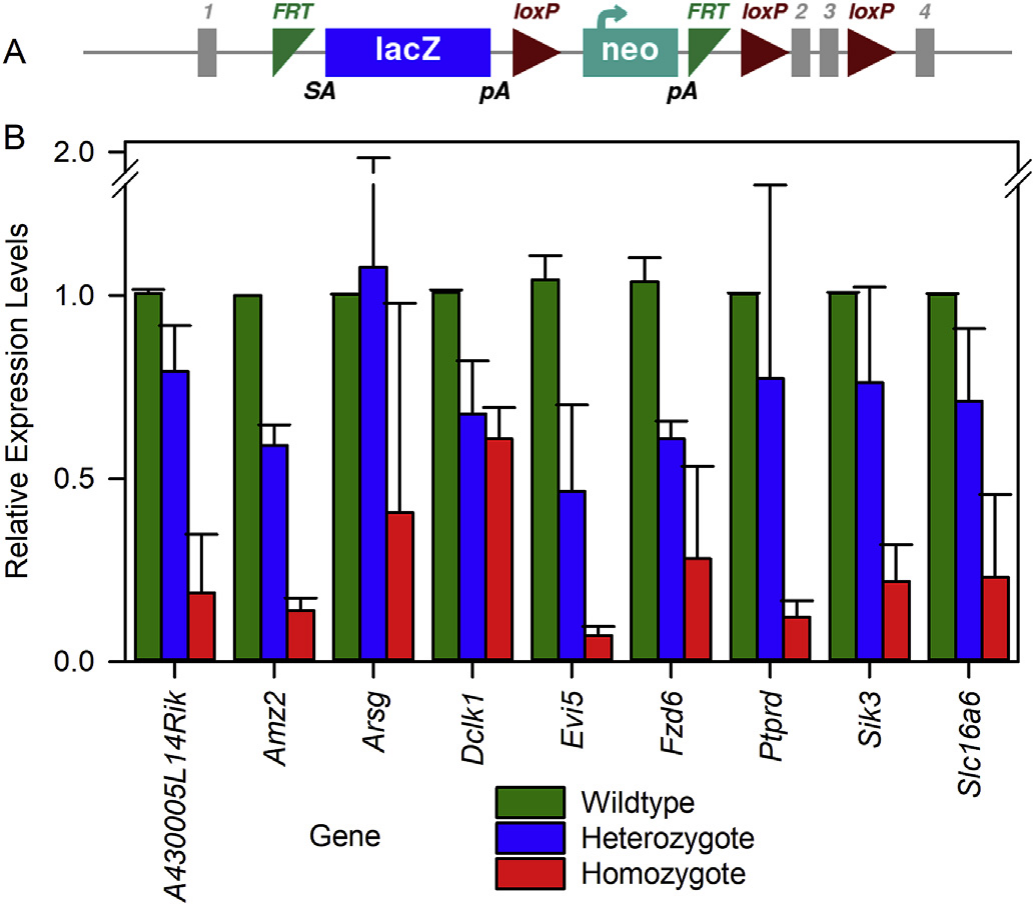
**A. A schematic representation of the gene targeting strategy used in this study.** In this case, the *A430005L14Rik*^*tm1a(KOMP)Wtsi*^ allele is illustrated. Gene expression is knocked down by insertion of a DNA cassette containing a lacZ reporter and neomycin (neo) resistance genes before the critical exon of the targeted gene (exon 2). This is a typical “tm1a” construct (see White et al., 2013) and is classed as knockout first, conditional-ready due to the addition of FRT and loxP recombinase sites around the insert cassette. **B. Relative gene expression levels in targeted mutant mice.** For 9 mutant lines, relative expression levels of mRNA are shown as mean ± SD for wildtype (green), heterozygote (blue) and homozygote (red) mice. Relative expression of each gene is normalised to wildtype levels (1.0). Data were obtained from the following numbers of mice for each gene (showing Wildtype, Heterozygote and Homozygote, respectively); *A430005L14Rik* 6,5,5; *Amz2* 1,5,5; *Arsg* 3,3,2; *Dclk1* 3,4,3; *Evi5* 3,3,4; *Fzd6* 4,3,5; *Ptprd* 3,4,3; *Sik3* 2,5,5; *Slc16a6* 4,4,4. (For interpretation of the references to color in this figure legend, the reader is referred to the Web version of this article.)

Mutant mice were created at the Wellcome Trust Sanger Institute on a C57BL/6N genetic background (White et al., 2013). Seventeen mutant mouse lines were investigated in this study. Of these, 15 carry a promoter-driven knockout-first allele, with a large cassette inserted in the intron before the targeted critical exon(s) which interferes with transcription leading to knockdown or knockout of expression (Fig. 1A). The inserted cassette contains the β-galactosidase/LacZ reporter gene (Skarnes et al., 2011; White et al., 2013). Two of the lines carry endonuclease-mediated exon deletion mutations. Table 2 details the alleles of each gene used in this study; further details can be found at www.mousephenotype.org. All mutant mice are available through the European Mouse Mutant Archive (EMMA). All mutant mice used were homozygotes for the mutation except for *Cmip* and *Pthlh* which are homozygous lethal, so we tested heterozygotes.

DNA extracted from ear-clips was used as the template for short range PCR, using custom primer sequences. For all lines, the mutant reaction used the gene-specific wild type forward primer (Table 3) paired with the reverse primer CasR1: TCGTGGTATCGTTATGCGCC which recognises the second FRT site in the cassette. Primers targeted against the neomycin gene in the cassette were used to determine the presence or absence of the cassette; forward- CAAGATGGATTGCACGCAGGTTCTC and reverse- GACGAGATCCTCGCCGTCGGGCATGCGCGCC. Further detail of genotyping methods can be found elsewhere (White et al., 2013).

**Table 3:** **Genotyping primer sequences**.

Gene	Allele	WT Forward Primer Sequence	WT Reverse Primer Sequence	Mutant Forward Primer Sequence	Mutant Reverse Primer Sequence
*A430005L14Rik*	*A430005L14Rik*^*tm1a(KOMP)Wtsi*^	TAAATAAAGGTGGGCGAGGC	ACCCAGCAGAACTGAGTGGAC	TAAATAAAGGTGGGCGAGGC	TCGTGGTATCGTTATGCGCC
*Amz2*	*Amz2*^*tm1e(KOMP)Wtsi*^	AGAAATACGTGTATTGGCATAGAA	TGCCGTAGTACCTGCATCTG	AGAAATACGTGTATTGGCATAGAA	TCGTGGTATCGTTATGCGCC
*Arsg*	*Arsg*^*tm1a(KOMP)Wtsi*^	TAGCGATTGAATGGCCTCAT	TTACCCAGCTATTTGTCCCTAA	TAGCGATTGAATGGCCTCAT	TCGTGGTATCGTTATGCGCC
*Dclk1*	*Dclk1*^*tm1a(EUCOMM)Wtsi*^	TCCAGAGTTTGAGACCTGTTGG	AGTCAGAGGAGGCACATCAGC	TCCAGAGTTTGAGACCTGTTGG	TCGTGGTATCGTTATGCGCC
*Evi5*	*Evi5*^*tm1a(KOMP)Wtsi*^	GGATTAAAGGTGTGTGCAGCC	TCTTCGTCTTCTTGGTAATTGCAG	GGATTAAAGGTGTGTGCAGCC	TCGTGGTATCGTTATGCGCC
*Fzd6*	*Fzd6*^*tm2a(EUCOMM)Wtsi*^	CGTCCATGCTGCTACACTGC	TGCATTCACAGATGTTGGGAG	CGTCCATGCTGCTACACTGC	TCGTGGTATCGTTATGCGCC
*Grm8*	*Grm8*^*tm2a(KOMP)Wtsi*^	TTCAAACTTTCGTGGCAAGC	AACAAATGAGAGGTCATGTCAACC	TTCAAACTTTCGTGGCAAGC	TCGTGGTATCGTTATGCGCC
*Ptprd*	*Ptprd*^*tm2a(KOMP)Wtsi*^	TCACCTCGCTGTTCTTCCTG	CTTCTCAGTGCCCAACCCTC	TCACCTCGCTGTTCTTCCTG	TCGTGGTATCGTTATGCGCC
*Sik3*	*Sik3*^*tm1a(EUCOMM)Hmgu*^	TGACTGCACCTTGAAAACGG	GCCCAAGTTTGTATGAGTGCC	TGACTGCACCTTGAAAACGG	TCGTGGTATCGTTATGCGCC
*Slc16a6*	*Slc16a6*^*tm1a(EUCOMM)Wtsi*^	CCCCATAAGCATGCCTCAA	CATCCTATTTAGCAAACATATCTGACA	CCCCATAAGCATGCCTCAA	TCGTGGTATCGTTATGCGCC
*Tgm6*	*Tgm6*^*tm1a(KOMP)Wtsi*^	CCCAGTGACAGCCACACAAG	GCCTGAAAATTAGGGCACTCTG	CCCAGTGACAGCCACACAAG	TCGTGGTATCGTTATGCGCC

Eleven mutant lines were analysed in detail using a battery of electrophysiological testing at 14 weeks old, comprising a basic hearing threshold characterisation by ABR and further measurements of auditory function including frequency tuning and temporal processing (see below). Homozygous mice were tested for each line (n = 6 minimum). Wildtype control mice from each line, littermates where possible, were also tested and their results pooled to form a large control dataset for each parameter under investigation. A further six lines were screened by measurement of ABR thresholds only, again comparing with a large set of wildtype mice on the same genetic background (Ingham et al., 2019).

### 2.4 Quantitative reverse-transcription PCR (qRT-PCR)

We assessed the degree of knockdown of expression of each gene by qRT-PCR, as described previously (Chen et al., 2014; Buniello et al., 2016). RNA was isolated from the whole brains of postnatal day (P)14 mice using TRI Reagent® (Sigma Aldrich) and reverse-transcribed into cDNA using Superscript II Reverse Transcriptase (Invitrogen, cat. no. 11904–018) after treatment with DNAse 1 (Sigma, cat no AMP-D1). Quantitative RT-PCR was carried out using Bio-Rad Master Mix (SsoFast and SsoAdvanced Master mixes, cat. nos 1725232, 1725281) and a gene-specific Taqman® expression assay (Life Technologies). Each sample was repeated in triplicate and the mean fold change between wildtype, heterozygous and homozygous mice replicate results was calculated using the 2^−ΔΔCΤ^ calculation (Livak and Schmittgen, 2001). The housekeeping gene *Hprt* was used as a reference to normalise the expression of the targeted gene in each mouse (Kang et al., 2013).

### 2.5 β-Galactosidase staining

We used the inserted β-galactosidase/LacZ reporter gene to indicate the location of expression of each gene, as described previously (Buniello et al., 2016; Chen et al., 2014). Inner ears were dissected from postnatal day (P)14 mice and fixed in 4% paraformaldehyde for 30–40 min with rotation at room temperature. Samples were washed with PBS twice for 20 min and decalcified in EDTA at 4 °C over 72 h. The inner ears were washed in detergent wash (2 mM MgCl_2_; 0.02% NP-40; 0.01% sodium deoxycholate in PBS, pH7.3) for 30 min at room temperature. X-gal (Promega, cat. no. V394A) was added 1:50 to pre-warmed staining solution (5 mM K_3_Fe(CN)_6_ and 5 mM K_4_Fe(CN)_6_ in detergent solution), and the samples were then incubated at 37 °C until each sample was deemed adequately stained. Following X-Gal staining, the samples were washed with PBS, dehydrated and embedded in paraffin wax. The samples were sectioned at 8  μm, counterstained using Nuclear Fast Red (VWR, cat. no. 342094W) and mounted using Eukitt quick-hardening mounting medium (Sigma-Aldrich) before being viewed and photographed using a Zeiss Axioskop microscope connected to an AxioCam camera and interfaced with Axiovision 3.0 software.

### 2.6 Auditory brainstem response (ABR) measurements

Mice were anaesthetised using 100 mg/kg Ketamine (Ketaset, Fort Dodge Animal Health) and 10 mg/kg Xylazine (Rompun, Bayer Animal Health) IP and positioned inside a sound attenuating chamber (Industrial Acoustics Company Limited, Model 400-A) on a homeothermic blanket at 20 cm distance from the sound delivery speaker. Subcutaneous needle electrodes (NeuroDart; UNIMED UK) were inserted on the vertex and overlying the left and right bullae (Ingham et al., 2011). Mice needed for repeated ABR recordings were given 1 mg/kg atipamezole (Antisedan, Pfizer) IP to promote recovery from the anaesthesia.

The mean (+/− SD) age (weeks) of wildtype mice was 14.3 ± 0.6 weeks (n = 37) and for mutant mice tested were: *A430005L14Rik*, 14.0 ± 0.1 (n = 9); *Amz2*, 14.2 ± 0.2 (n = 6); *Arsg*, 14.1 ± 0.1 (n = 6); *Dclk1*, 14.0 ± 0.5 (n = 6); *Evi5*, 13.9 ± 0.5 (n = 7); *Fzd6*, 14.3 ± 0.5 (n = 6); *Grm8*, 14.3 ± 0.4 (n = 6); *Ptprd*, 14.2 ± 0.1 (n = 7); *Sik3*, 14.6 ± 0.3 (n = 8); *Slc16a6*, 14.4 ± 0.4 (n = 6); *Tgm6*, 14.0 ± 0.3 (n = 8); *Cmip*, 14.1 ± 0.2 weeks (n = 8 heterozygotes), 14.0 ± 0.2 weeks (n = 8 wildtypes); *Csmd1*, 13.9 ± 0.2 weeks (n = 4 homozygotes), 14.1 ± 0.1 weeks (n = 12 wildtypes); *Dipk1a*, 13.9 ± 0.2 weeks (n = 6 homozygotes), 14.0 ± 0.2 weeks (n = 6 wildtypes); *Optn*, 14.0 ± 0.1 weeks (n = 9 homozygotes), 14.0 ± 0.2 weeks (n = 14 wildtypes); *Pthlh*, 13.9 ± 0.0 weeks (n = 4 heterozygotes), 13.9 ± 0.2 weeks (n = 10 wildtypes); *Rimbp2*, 14.0 ± 0.1 weeks (n = 8 homozygotes), 14.0 ± 0.2 weeks (n = 10 wildtypes).

Free-field acoustic stimulation and recording of neural activity was controlled via a custom software application interacting with a TDT RZ6 multifunction processor (Tucker Davis Technologies, Alachua, USA). Stimuli were synthesised in software, uploaded to a digital buffer on the RZ6 processor and attenuated to the required sound level when the stimuli were presented through a TDT FF1 loudspeaker, using an equalisation curve generated by Fast Fourier Transformation of the signal recorded on a ¼” condenser microphone (PCB Piezotronics, Depew, New York USA; Model 377C01 microphone, 426B03 preamplifier & 480C02 signal conditioner) placed at 20 cm distance following presentation of a broadband noise stimulus.

Evoked EEG potentials picked up by the needle electrodes were amplified and digitized within the sound chamber using a TDT RA4LI low impedance headstage and RA4PA preamplifier, before being returned to the RZ6 optical interface via a fibreoptic cable. The electrode input was amplified and bandpass filtered to 300–3000 Hz. Under software control, snippets of filtered EEG signal, synchronised to stimulus presentation, were averaged in a digital buffer on the RZ6 processor before being returned to the software application.

To measure threshold sensitivity, we presented click stimuli (10 μs duration) and tone pips (5 m s duration, with a 1 m s onset and offset ramp) of 3, 6, 12, 18, 24, 30, 36 & 42 kHz over sound levels ranging from 0 to 95 dB SPL in 5 dB increments (detailed in Ingham et al., 2011), at a fixed presentation rate of 42.6 stimuli per second. ABRs were recorded as an average of 256 presentations of each stimulus, 20 m s in duration, triggered by the same digital signal which triggered acoustic stimuli. Responses were stacked from low to high stimulus level to allow visual determination of threshold, defined as the lowest stimulus level which evoked a response waveform with peak, trough or slope features consistent with the trends of these features at higher stimulus levels, determined by visual inspection. Growth functions of ABR wave 1 peak-peak amplitude as a function of sound pressure level were analysed.

#### 2.6.1 Frequency tuning curves

ABR wave 1 amplitudes were used to plot frequency tuning curves with a forward masking paradigm. A probe tone (either 12 kHz, 18 kHz or 24 kHz, 5 m s duration, 1 m s rise/fall time, presented at threshold +20 dB) was presented with a 4 m s gap after a masker tone of variable frequency (in a ratio of 0.5, 0.7, 0.9, 0.95, 1.0, 1.05, 1.1, 1.3, or 1.6 relative to the probe frequency), 10 m s duration, with a 1 m s rise/fall time, presented at levels ranging from 0 to 90 dB SPL in 10 dB steps. For each probe tone, masked threshold was estimated for each masker frequency as the masker level that resulted in a 3 dB (50% magnitude) reduction in ABR wave 1 amplitude and plotted as a function of masker frequency to produce 3 frequency tuning curves for each mouse.

#### 2.6.2 Adaptation using forward masking

As a measure of the depth of adaptation of response and of the time course of recovery from this adaptation, we measured click-evoked ABR wave 1 amplitudes in a forward masking stimulus paradigm. Stimuli consisted of a 10-ms burst of masking white noise, followed by a probe click presented at intervals of 4, 8, 16, 32 & 64 m s after the noise burst and a reference click presented 150 m s after the onset of the noise burst. Click stimuli were presented at 20 dB or 50 dB above click threshold, with the masking noise presented at 10 dB lower than the click level. The amplitude of the ABR wave 1 in response to the probe click was measured and normalised to the amplitude of the response to the reference click, then plotted against masker-probe gap size.

#### 2.6.3 Adaptation to stimulus repetition rate

To assess adaptation of auditory nerve firing, we measured ABR wave 1 amplitude as a function of sound level and stimulus presentation rate. We used click stimuli, presented from 0 to 95 dB SPL in 5 dB steps in randomised order. Each ABR was recorded as an average of 256 presentations of a particular level of the click stimulus, before moving onto the next randomly selected dB SPL. Multiple level series were generated, recorded at increasing presentation rates from 10.65, 21.3, 42.6 and 85.2 clicks/second. ABR wave 1 amplitude was measured and plotted as function of dB SPL to form growth curves at each presentation rate.

#### 2.6.4 Desynchronization of auditory nerve firing

To assess desynchronization of discharge, we measured ABR wave 1 amplitude as a function of sound level and as a function of the ramp time of an 18 kHz 8 m s duration stimulus. Tone stimuli were presented from 0 to 95 dB SPL, in 5 dB steps, in randomised order. Each ABR was recorded as an average to 256 presentations of a particular level of the stimulus, before moving onto the next randomly selected dB SPL. Multiple level series were generated, recorded at increasing tone ramp time, from 0.25, 0.50, 1.0, 2.0 and 4.0 m s ramp times. Evoked ABR wave 1 amplitude was measured and plotted as a function of dB SPL to form growth curves for each ramp time.

### 2.7 Susceptibility to noise damage

Sex-matched littermate pairs were placed into separate compartments of a wire mesh cage suspended in the middle of a noise-exposure chamber designed with no parallel surfaces to minimise any reverberation (Holme and Steel, 2004). Broadband noise was generated via a TDT RZ6 multifunction processer, using RPvdsEX software. The noise was bandpass filtered to an octave band of 8–16 kHz, digitally using the RZ6 processor before digital – analogue conversion, amplification with a Bruel & Kjaer Type 2716c power amplifier, and presented to the mice inside the chamber via a compression driver (JBL 2446H, Northridge, CA) connected to a flat front biradial horn (JBL 2380A, Northridge, CA) secured to the roof of the sound box. Sound pressure level was controlled using RPvdsEX software and was measured above the mouse cage using a Bruel & Kjaer Type 4938 microphone and Type2670 preamplifier connected via a Type 3560-C chassis & Type 7536 controller module to a Type 3110 Input-Output Module driven by Bruel & Kjaer PULSE X software. Presentation of the noise started at approximately 66 dB SPL and was stepped up to 100 dB SPL over 2 min where it was maintained for 60 min before being stepped down over a 2 min period. Mice continued to explore their environment as normal throughout the exposure period and no audiogenic seizures were seen.

ABRs were recorded from all noise-exposed mutant (*A430005L14Rik*, n = 11; *Grm8*, n = 11) and wildtype mice (n = 10) and sham-exposed (no noise delivered) control mice (n = 10) at least 1 day before their time in the noise-exposure box, followed by testing at 1 day, 3 days, 7 days and 14 days after the noise-exposure. They were then subjected to the full battery of auditory tests described above at 14 weeks old. Sham-exposed control mice were pooled from the *A430005L14Rik* (n = 6) and *Grm8* (n = 4) mutant lines and were heterozygotes.

### 2.8 Endocochlear potential

Endocochlear potential (EP) was measured in urethane-anaesthetised (0.1 ml/10 g bodyweight of a 20%w/v solution) mice from the *Dclk1* mutant line aged 12 months using 150 mM KCl filled glass pipette microelectrodes. EP was recorded as the potential difference between the tip of a glass microelectrode when inserted into scala media via a fenestration in the cochlea basal turn lateral wall and a reference Ag–AgCl pellet electrode inserted under the skin of the dorsal surface of the neck (See Steel and Barkway, 1989; Chen et al., 2014; Ingham et al., 2016). Endocochlear potential was measured in 12 month old wildtype (n = 4), heterozygote (n = 2) and homozygote (n = 2) *Dclk1* mice.

### 2.9 Statistical analyses

As all mice had the same genetic background, we pooled results for each test obtained from wildtype mice across all lines tested and used a Bonferroni-corrected significance level for each comparison. In all cases, data did not conform to a normal distribution (Shapiro-Wilk test) and were compared using the Kruskall Wallis non-parametric Analysis of Variance (ANOVA), with Dunn’s method for multiple comparisons vs a control group. All statistical tests were carried out using SigmaPlot v13.0 (Systat Software, Inc.).

Mean forward masking curves were fitted with an exponential growth to maximum function (equation (1)) using SigmaPlot v13.0. This function fitted the mean data well, with R^2^ values ranging from 0.976 to 0.999 across the 12 functions (mean R^2^ +/− SD = 0.990 ± 0.007).

(1) y = y_0_ + a(1-e^-bx^)

where.

y_0_ = predicted amplitude at 0 m s gap (when x = 0 m s),
a = maximum y-value,
1/b = tau, the time constant of recovery.

## 3 Results

### 3.1 Mutant alleles led to knockdown of expression

Relative levels of mRNA expression in each of the mutant lines examined here are shown in Fig. 1. Expression levels within each line were normalised to a value of 1 for wildtype mice (green bars), with heterozygotes (blue) and homozygous mutants (red) mean relative expression levels plotted. All 9 lines tested showed a variable degree of knockdown of mRNA levels (to 7.2%–60.8% of wildtype levels). For most of the lines, other abnormal phenotypes were found in the course of a high-throughput screen (White et al., 2013; Ingham et al., 2019; International Mouse Phenotyping Consortium, https://www.mousephenotype.org/; see summary in Table 2), suggesting that the knockdown of gene expression was enough to affect gene function. For two of the lines, we generated the *tm1b* allele by crossing the *tm1a* allele to a Cre recombinase-expressing line on the same genetic background in order to delete a critical exon and produce a null allele (Skarnes et al., 2011; Chen et al., 2014). The *Dclk1*^*tm1b*^ mutants showed the same auditory phenotype as in *Dclk1*^*tm1a*^ so were not followed further (data not shown). The *Sik3*^*tm1a*^ allele was also converted to the *Sik3*^*tm1b*^ allele by exposure to Cre recombinase, but the new allele was homozygous lethal, so we were not able to assess auditory function in this line. For *Sik3*^*tm1a*^ we obtained 26 homozygous mutants, 53 heterozygotes and 21 wildtypes from heterozygote by heterozygote matings which fits a Mendelian pattern of inheritance, while from the *Sik3*^*tm1b*^ allele, we obtained 40 heterozygotes and 26 wildtypes but no homozygotes.

### 3.2 Targeted genes were expressed in the cochlea

**Fig. 2:**
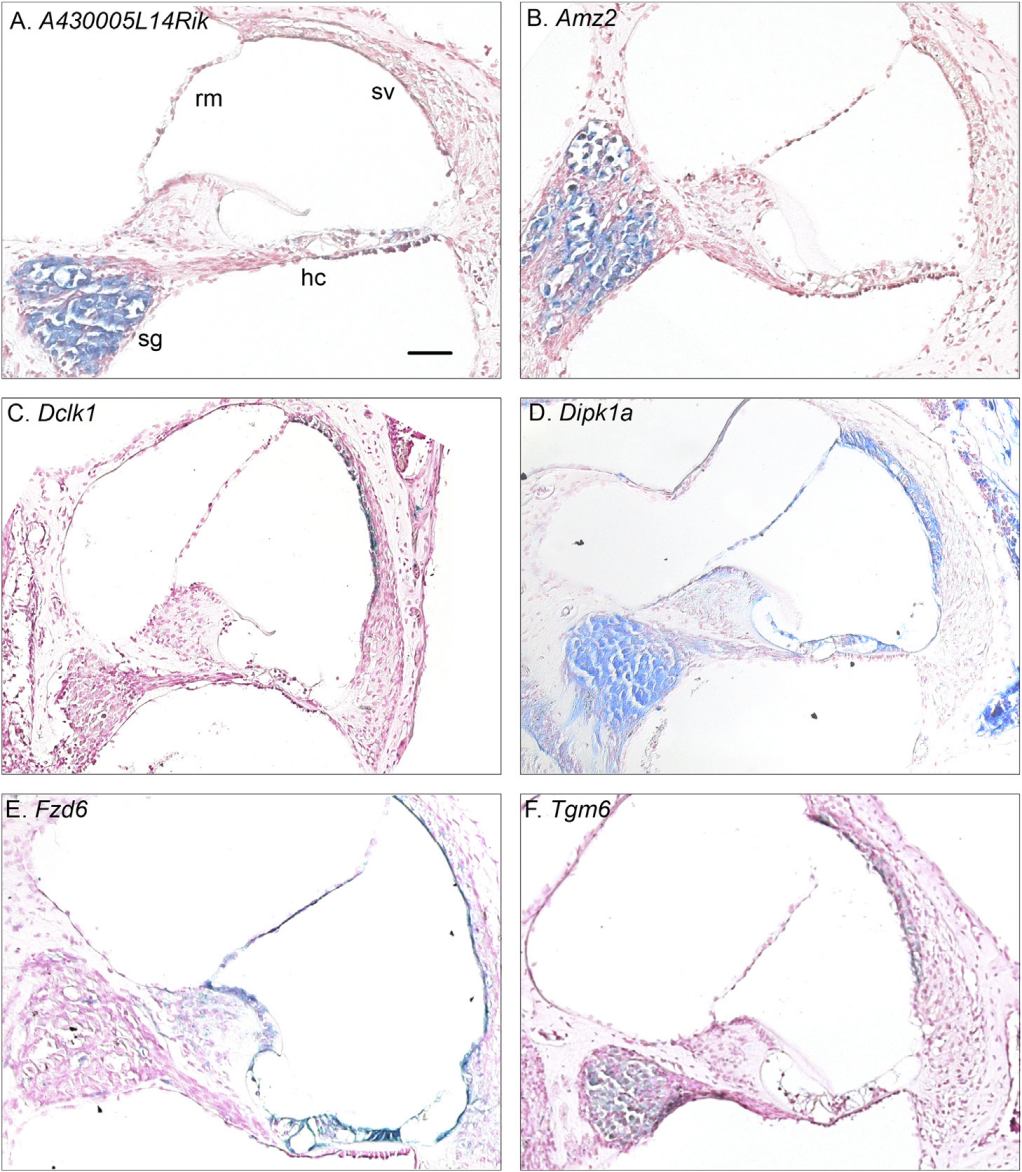
**LacZ expression in a selection of mutant lines.** Here, we illustrate LacZ expression, visualised by X-Gal staining (blue reaction product) against the pink Nuclear Fast Red counterstaining, in 6 of the mutant lines examined in this study. *A430005L14Rik* (A) and *Amz2* (B) are predominantly expressed in the spiral ganglion. *Dclk1* (C) showed clearest expression in the stria vascularis. *Dipk1a* (D) was expressed in epithelial cells lining the scala media and in the spiral ganglion. *Fzd6* (E) was widely expressed throughout epithelial cells bordering scala media. *Tgm6* (F) was expressed in the stria vascularis and spiral ganglion. For reference, some major structures visible in each section are labelled in panel (A); Hair Cells & the organ of Corti, hc; Stria Vascularis, sv; Reissner’s Membrane, rm; Spiral Ganglion, sg. The scale bar shown in panel (A) indicates 100  μm and applies to each image.

Gene expression patterns in the cochlea were investigated using the LacZ reporter system to detect expression of the β-galactosidase reporter gene contained in each mutant line. Some examples of expression patterns are shown in Fig. 2 and are summarised in Table 2 for each of the genes examined in this study. The patterns of mRNA expression are broadly similar to the immunolocalization previously published for some of these genes (Girotto et al., 2014; Wolber et al., 2014).

### 3.3 Mutant mice showed normal thresholds and waveforms

**Fig. 3:**
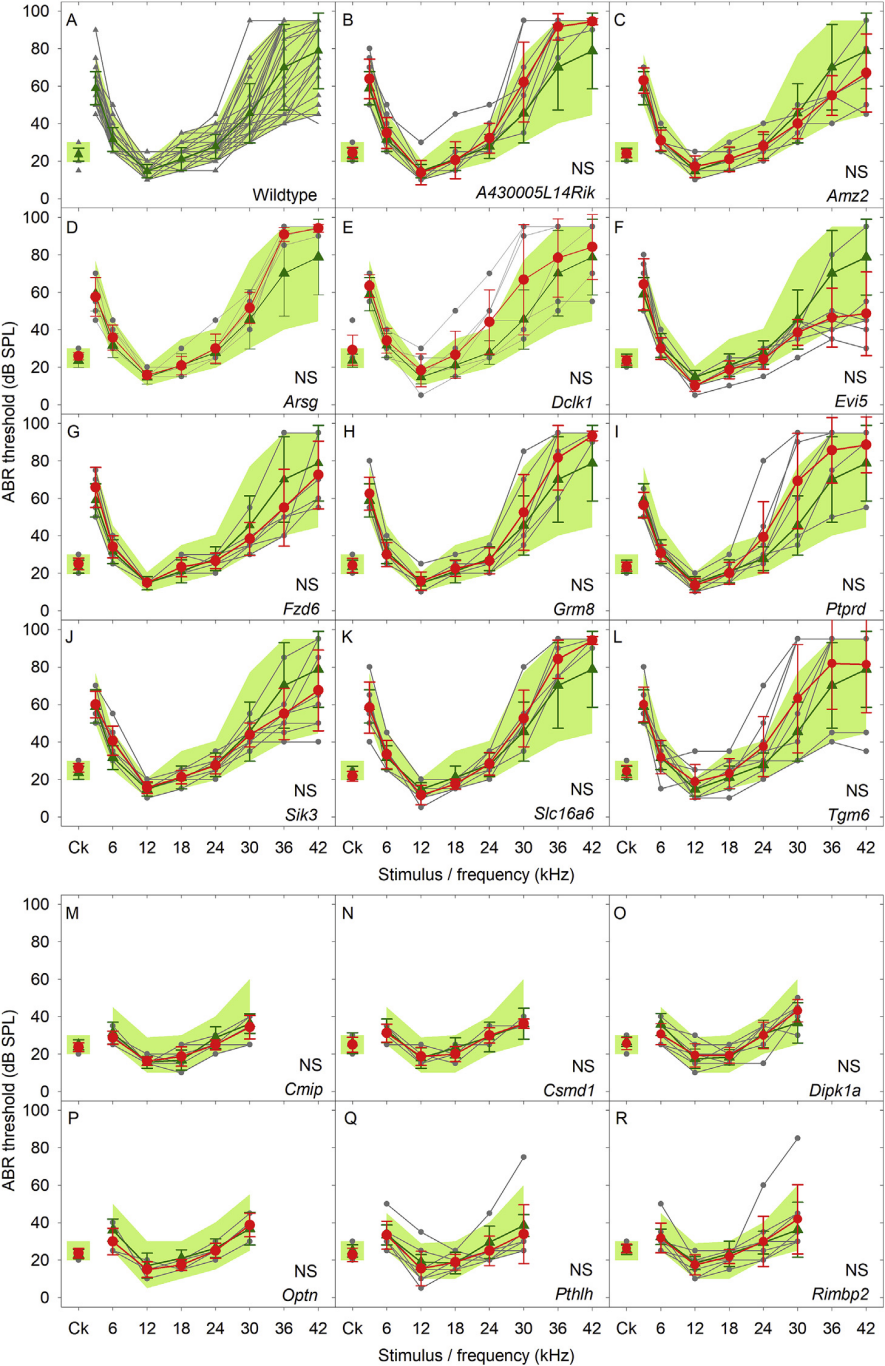
**ABR thresholds in wildtype and mutant mice.** A. ABR thresholds measured in 37 wildtype mice are plotted as grey triangles. These were used to generate a 95% reference range for normal thresholds, spanning the 2.5 percentile to 97.5 percentile of thresholds recorded for each stimulus plotted as a green area. The wildtype mean threshold (+/− SD) for each stimulus is plotted as green triangle symbols and lines. B-L. Comparison of wildtype and mutant thresholds. In each panel, the reference range of wildtype thresholds, and the mean (+/− SD) wildtype threshold are replotted. Results from individual mutants are plotted as filled grey circles and lines, with the mutant mean threshold (+/− SD) plotted as red circles and lines. Using Kruskall-Wallis non-parametric ANOVA with a Bonferroni-corrected significance level of 0.00417 (0.05/12 test groups), none of the mutant groups had thresholds significantly different to the control values (ANOVA p = 0.142, indicated by NS; see also Table 4). M-R indicate ABR thresholds recorded as part of a high throughput phenotyping screen (Ingham et al., 2019) for mice carrying targeted mutations in *Cmip* (M), *Csmd1* (N), *Dipk1a* (O), *Optn* (P), *Pthlh* (Q) and *Rimbp2* (R). None of these mutant groups had thresholds significantly different to the control reference range (indicated by NS). (For interpretation of the references to color in this figure legend, the reader is referred to the Web version of this article.)

Fig. 3 plots ABR thresholds recorded in the mice aged 14 weeks. There were no significant differences between any of the 11 mutant groups studied in detail here (Fig. 3B-L) compared to the wildtype controls (Fig. 3A, Table 4). The further 6 mutant lines of interest are also plotted here for comparison. ABR Thresholds from these 6 lines (Fig. 3M–R) were not significantly different from wildtype control thresholds (as listed in Ingham et al., 2019).

**Table 4:** **Statistical data associated with physiological test.** Comparisons were made using Dunn’s Method of multiple comparisons (vs a control group), as a post-hoc test following Kruskall-Wallis One Way ANOVA on ranks. Q, the ‘Q’ result of Dunn’s method. p, the p-value resulting from the test. Statistically significant results are indicated in **bold text**, where the p-value is less than the Bonferroni-corrected significance level for the test group (0.00417, or 0.0125 for noise-exposure groups).

Allele	Comparison		ABR Threshold	Freq Tuning Curves			Forward Masking		ABR wave 1 adaptation				ABR wave 1 desynchronization				
				Probe Tone Masked Threshold			P1N1 Amp Ratio		P1N1 Amp				P1N1 Amp				
				12 kHz	18 kHz	24 kHz	+ 20 dB	+ 50 dB	10.7/s	21.3/s	42.6/s	85.2/s	0.25 m s	0.5 m s	1 m s	2 m s	4 m s
*A430005L14Rik*	vs WT	Q	1.66	0.39	0.27	0.30	0.35	0.52	0.07	0.52	0.41	0.66	0.88	0.52	0.75	2.07	0.45
		p	1	1	1	1	1	1	1	1	1	1	1	1	1	0.423	1
*Amz2*	vs WT	Q	0.15	0.52	0.07	0.68	0.22	0.14	1.87	0.29	2.33	2.48	3.13	2.67	2.87	2.78	3.03
		p	1	1	1	1	1	1	0.673	1	0.216	0.144	0.019	0.084	0.046	0.061	0.027
*Arsg*	vs WT	Q	1.15	0.47	0.47	0.46	0.48	0.39	1.00	0.09	1.55	1.42	2.29	1.82	2.06	2.42	1.18
		p	1	1	1	1	1	1	1	1	1	1	0.242	0.758	0.435	0.171	1
*Dclk1*	vs WT	Q	2.17	0.98	0.01	1.46	0.42	0.02	0.87	2.75	0.17	0.17	1.55	1.60	1.10	1.24	1.89
		p	0.33	1	1	1	1	1	1	0.066	1	1	1	1	1	1	0.65
*Evi5*	vs WT	Q	1.82	1.37	0.35	0.93	0.58	0.88	2.70	1.16	2.75	2.44	2.39	2.48	2.42	1.35	1.90
		p	0.758	1	1	1	1	1	0.077	1	0.066	0.163	0.188	0.146	0.172	1	0.632
*Fzd6*	vs WT	Q	0.05	0.74	0.22	0.34	0.69	0.38	0.20	1.37	1.16	0.69	0.98	1.05	1.28	0.77	0.17
		p	1	1	1	1	1	1	1	1	1	1	1	1	1	1	1
*Grm8*	vs WT	Q	0.64	0.32	0.57	1.48	2.06	0.70	2.18	2.91	1.89	1.40	3.22	3.39	2.97	3.22	3.07
		p	1	1	1	1	0.438	1	0.322	0.04	0.642	1	0.014	0.008	0.033	0.014	0.023
*Ptprd*	vs WT	Q	1.176	0.161	0.309	0.814	1.079	0.4	0.345	0.0782	0.241	0.508	1.41	1.203	1.61	1.926	1.965
		p	1	1	1	1	1	1	1	1	1	1	1	1	1	0.596	0.544
*Sik3*	vs WT	Q	0.25	0.95	1.92	0.27	1.04	1.52	0.08	1.40	0.86	1.10	0.99	1.06	1.38	1.30	1.76
		p	1	1	0.6	1	1	1	1	1	1	1	1	1	1	1	0.855
*Slc16a6*	vs WT	Q	0.29	0.80	0.63	0.32	0.90	0.42	0.35	1.23	0.08	0.57	1.07	0.93	0.73	0.07	0.54
		p	1	1	1	1	1	1	1	1	1	1	1	1	1	1	1
*Tgm6*	vs WT	Q	1.46	0.82	0.28	1.24	0.42	0.36	0.11	1.35	0.24	0.37	0.29	0.61	0.92	0.31	1.14
		p	1	1	1	1	1	1	1	1	1	1	1	1	1	1	1
Noise-Exposed																	
WT	vs SHAM	Q	2.29	0.41	1.77	1.31	0.97	0.68	0.22	0.28	1.02	1.67	0.61	1.74	1.82	1.15	0.53
		p	0.067	1	0.229	0.571	0.996	1	1	1	0.92	0.283	1	0.248	0.207	0.755	1
*A430005L14Rik*	vs SHAM	Q	**3.66**	1.77	**3.89**	1.29	0.37	0.39	**3.31**	2.66	2.04	**3.48**	**4.22**	**5.64**	**6.26**	**5.96**	**4.39**
		p	<**0.001**	0.232	<**0.001**	0.596	1	1	**p** = **0.007**	0.023	0.125	**0.001**	**p < 0.001**	**p < 0.001**	**p < 0.001**	**p < 0.001**	**p < 0.001**
*A430005L14Rik*	vs WT	Q	1.32	2.19	2.22	0.23	1.44	1.15	**3.47**	**3.14**	**3.27**	**3.48**	**3.70**	**3.95**	**4.51**	**4.92**	**5.12**
		p	0.56	0.086	0.079	1	0.449	0.756	**p** = **0.002**	**p** = **0.005**	**p** = **0.003**	**p** = **0.001**	**p < 0.001**	**p < 0.001**	**p < 0.001**	**p < 0.001**	**p < 0.001**
*Grm8*	vs SHAM	Q	1.87	0.64	1.93	1.53	0.54	1.54	0.50	0.47	1.11	1.20	1.04	2.12	2.35	1.84	0.19
		p	0.186	1	0.16	0.379	1	0.37	1	1	0.795	0.691	0.902	0.102	0.057	0.198	1
*Grm8*	vs WT	Q	0.47	1.06	0.12	0.15	0.48	0.90	0.30	0.19	0.08	0.54	0.41	0.32	0.46	0.66	0.77
		p	1	0.865	1	1	1	1	1	1	1	1	1	1	1	1	1

**Fig. 4:**
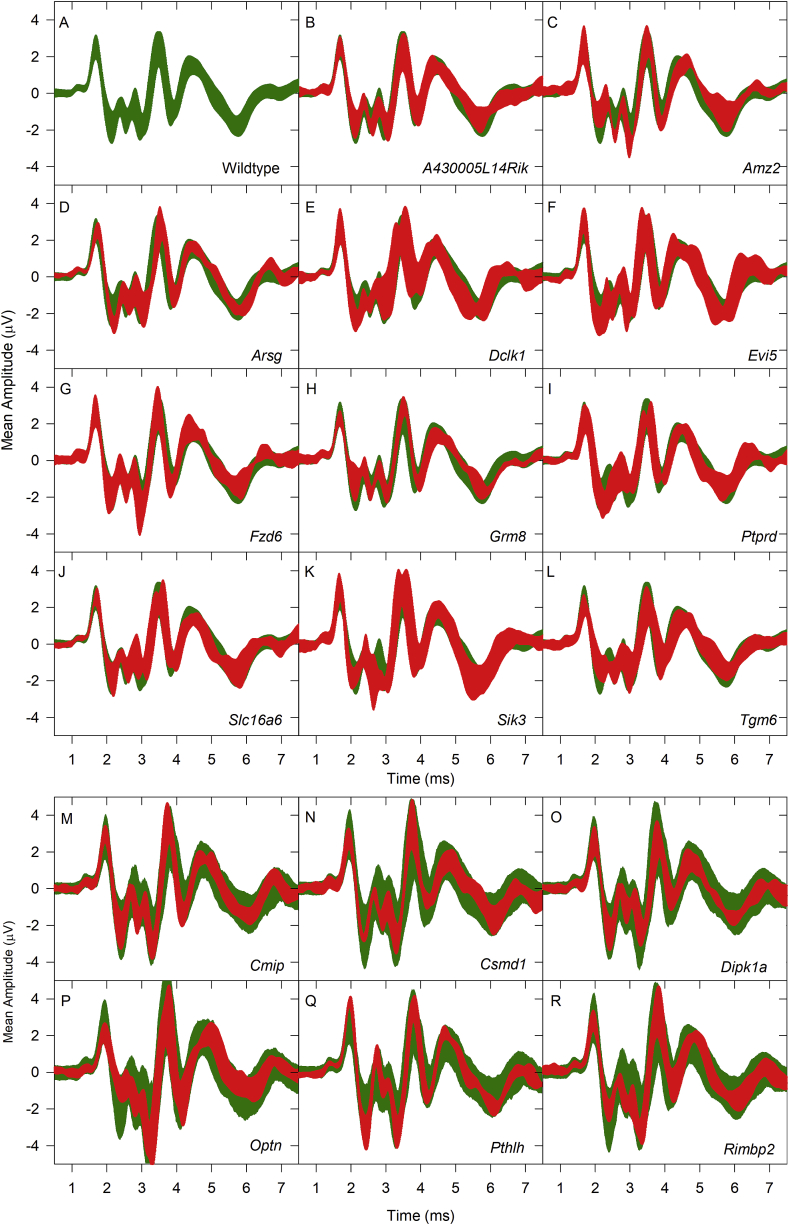
**ABR Waveform shape in wildtype and mutant mice.** Comparisons are made of click-evoked ABR waveform shapes recorded at 50 dB SL (sensation level, dB above threshold). For the wildtype group and each mutant group, the mean ABR amplitude across all mice in each group was calculated. This is plotted as a band representing the mean amplitude ± standard deviation (SD). A. 50 dB SL click-evoked ABR waveforms recorded from wildtype mice are plotted in green. In panels B–L, the wildtype data are replotted, along with the mean (+/− SD) amplitude for the mutant mice (in red), labelled on each panel. M-R indicate ABR thresholds recorded as part of a high throughput phenotyping screen (Ingham et al., 2019) for mice carrying targeted mutations in *Cmip* (M), *Csmd1* (N), *Dipk1a* (O), *Optn* (P), *Pthlh* (Q) and *Rimbp2* (R), plotted as mean (+/− SD) amplitude (in red) for each mutant line. In M-R, the green area represents a 2.5%ile-97.5%ile (95%) reference range of amplitude recorded from a large population of wildtype mice. (For interpretation of the references to color in this figure legend, the reader is referred to the Web version of this article.)

Fig. 4 plots mean click-evoked ABR waveforms (at 50 dB sensation level) calculated from responses obtained in the wildtype group (Fig. 4A) and the 11 mutant groups (Fig. 4B-L) tested. All mutant ABR waveforms were similar to their littermate controls; the overall gross features of the waveforms (peak-to-peak amplitudes, latencies, etc.) are similar in each case.

### 3.4 Frequency tuning was normal in mutant mice

**Fig. 5:**
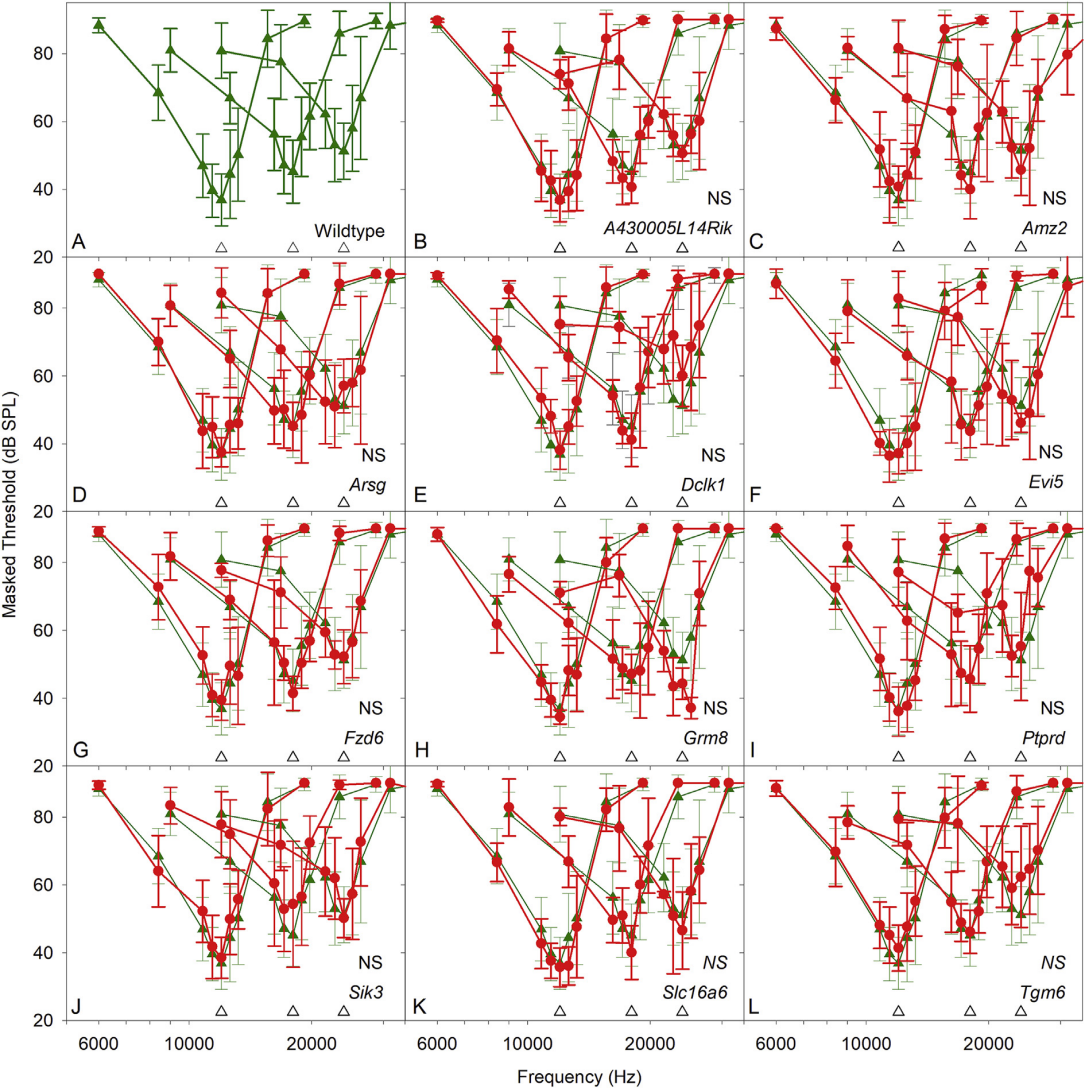
**Frequency Tuning Curves in wildtype and mutant mice.** Each panel illustrates Frequency Tuning Curves generated using probe tones set at 20–25 dB sensation level, at 12 kHz, 18 kHz and 24 kHz, indicated by the upward pointing open triangles positioned close to the frequency axis. Mean masked threshold (+/− SD) is plotted as a function of the masker frequency in each case. A. Masked thresholds measured across wildtype mice are shown in green (triangle symbols). B-L. Comparisons of wildtype and mutant masked thresholds. In each panel, the wildtype data are replotted from panel A for comparison to the data measured in mutant mice, shown in red (circle symbols, mean ± SD). (NS) indicates no significant differences in the masked threshold tuning curves of 14 week old mice (see also Table 4). (For interpretation of the references to color in this figure legend, the reader is referred to the Web version of this article.)

Frequency tuning curves were generated for wildtype and mutant mice, using a forward masking paradigm, with probe frequencies at 12 kHz, 18 kHz and 24 kHz, to span the more sensitive regions of the mouse audiogram (Fig. 5). There were no significant differences in tuning curves between the wildtype group and any of the mutant groups (Table 4).

### 3.5 No evidence of temporal processing defects in mutant mice

**Fig. 6:**
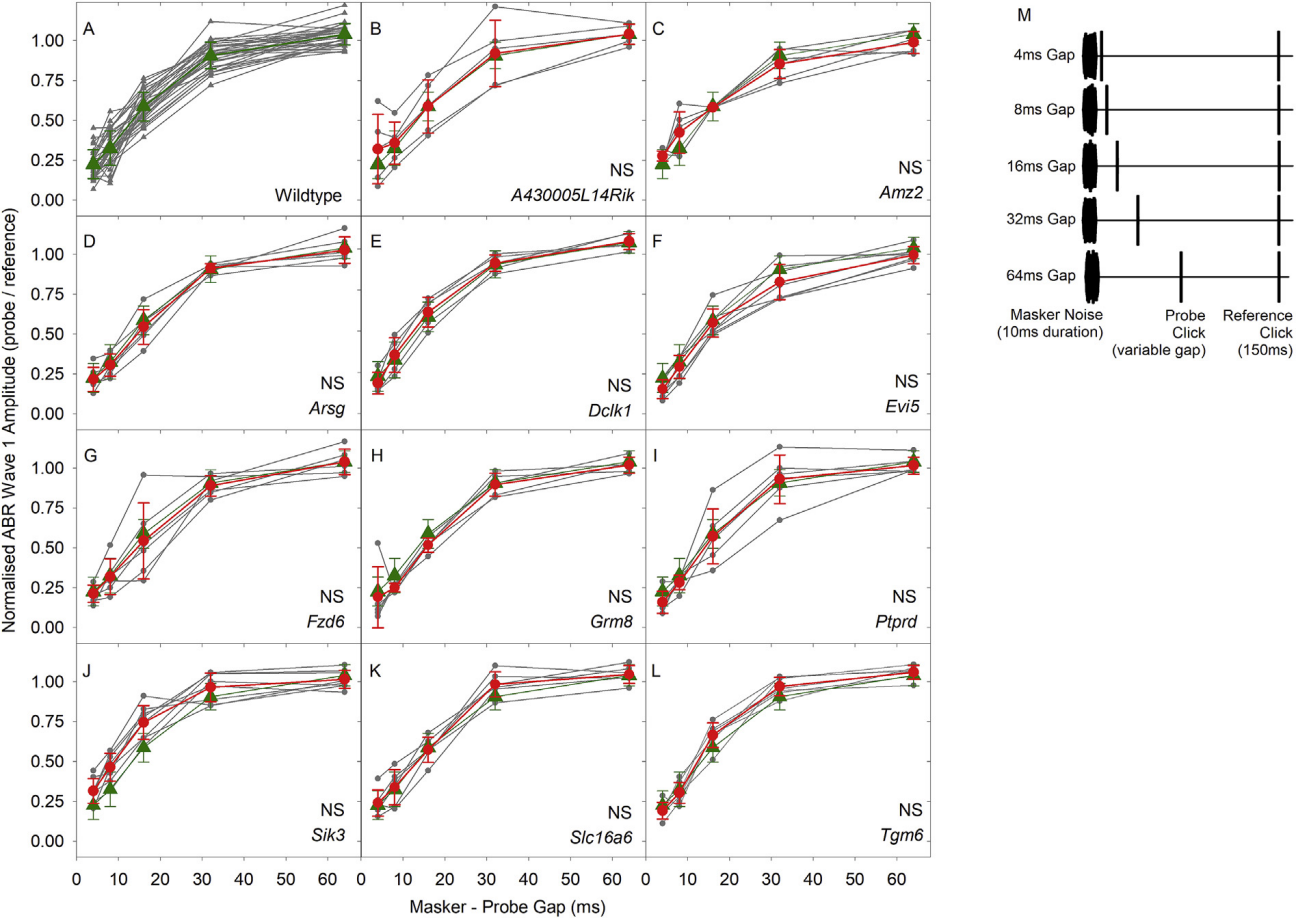
**Forward Masking in wildtype and mutant mice.** A. Forward Masking and Recovery functions measured in wildtype mice. ABR Wave 1 amplitude evoked by a probe click placed at variable gaps afters a 10 m s masker noise burst, normalised to wave 1 amplitude of a reference click (mean ± SD) is plotted as a function of the masker – probe gap. Results from individual mice are plotted in grey. The wildtype mean normalised wave 1 amplitudes (+/− SD) are plotted in green (triangles). B-L. Comparison of wildtype and mutant forward masking functions. In each panel, the mean (+/− SD) wildtype forward masking functions, as described for panel A, are replotted for comparison to the functions measured in mutant mice. In each of panels B–L, results from individual mutants are plotted in grey, with the mutant mean threshold (+/− SD) plotted in red circles and lines. (NS) indicates no significant differences in the forward masking curves of 14 week old mice (see also Table 4). M. The stimulus presentation scheme. (For interpretation of the references to color in this figure legend, the reader is referred to the Web version of this article.)

To assess recovery of wave 1 amplitude from a preceding sound, we generated forward masking functions for wildtype and mutant mice using probe and reference clicks presented at 20 dB and 50 dB sensation level (SL, dB above click threshold). Fig. 6 plots these functions for the 50 dB SL stimuli. There were no significant differences between the wildtype group and any of the mutant groups for either 20 dB SL or 50 dB SL (Table 4). ABR wave 1 amplitude evoked by a probe click with a short gap following a masker noise burst was reduced and this amplitude recovered with increasing gap duration to reach complete recovery with a 64 m s gap, following an exponential growth function (Fig. 6A; see methods). The maximal adaptation of wave 1 amplitude predicted by these fits (y_0_) ranged from −0.115 to 0.122 with a mean (+/− SD) of −0.009 ± 0.077. The time constant of recovery of wave 1 amplitude predicted by these fits ranged from 13.2 m s to 23.8 m s with a mean (+/− SD) of 19.6 m s ± 3.1 m s. Maximal recovery (a), predicted from the curve fits, ranged from 0.92 to 1.22 with a mean (+/− SD) of 1.10 ± 0.08.

**Fig. 7:**
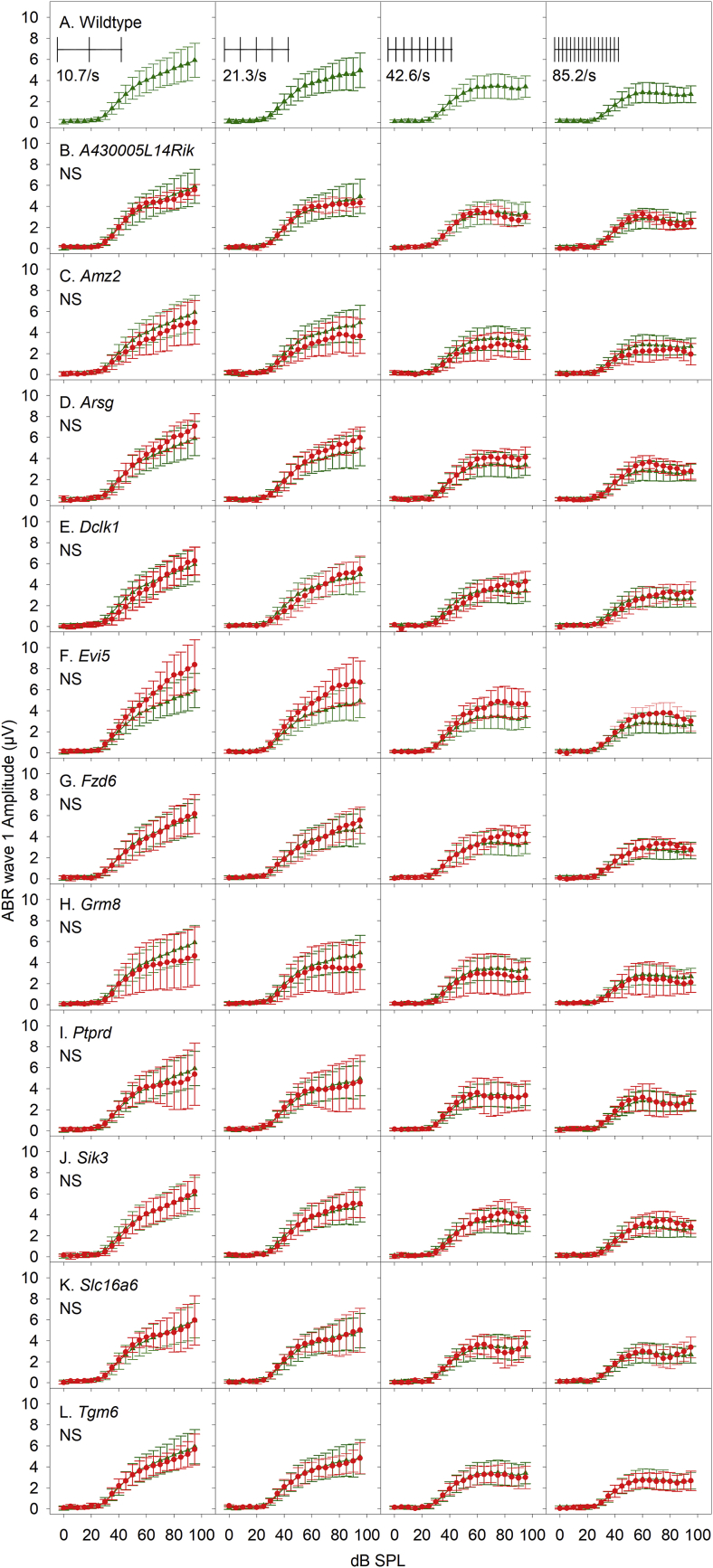
**Responses to increasing stimulus presentation rate.** Each panel plots mean ABR wave 1 amplitude as function of stimulus level (dB SPL). Responses to click stimuli presented at increasing rates from 10.7, 21.3, 42.6 and 85.2/sec are plotted in columns from left-right across the figure. The stimuli are illustrated schematically in the inset panels of row A. Row A plots mean (+/− SD) wave 1 amplitude from wildtype in green (triangles). Rows B-L replot this wildtype data and also plot equivalent data recorded from each mutant line in red (circles). (NS) indicates there is no statistically significant difference between the curves plotted (see also Table 4). (For interpretation of the references to color in this figure legend, the reader is referred to the Web version of this article.)

A second approach to assess wave 1 recovery was used, by increasing stimulus repetition rates. Using click stimuli presented at over an 8-fold range of rates, from 10.65 to 85.2 clicks/s, to produce increasing amounts of adaptation of neural firing rate, we generated ABR wave 1 amplitude growth curves to stimuli ranging from 0 to 95 dB SPL in 5 dB increments (Fig. 7). There were no significant differences in the distribution of wave 1 amplitude across any of the click presentation rates tested between the wildtype group or any of the mutant groups (Table 4).

**Fig. 8:**
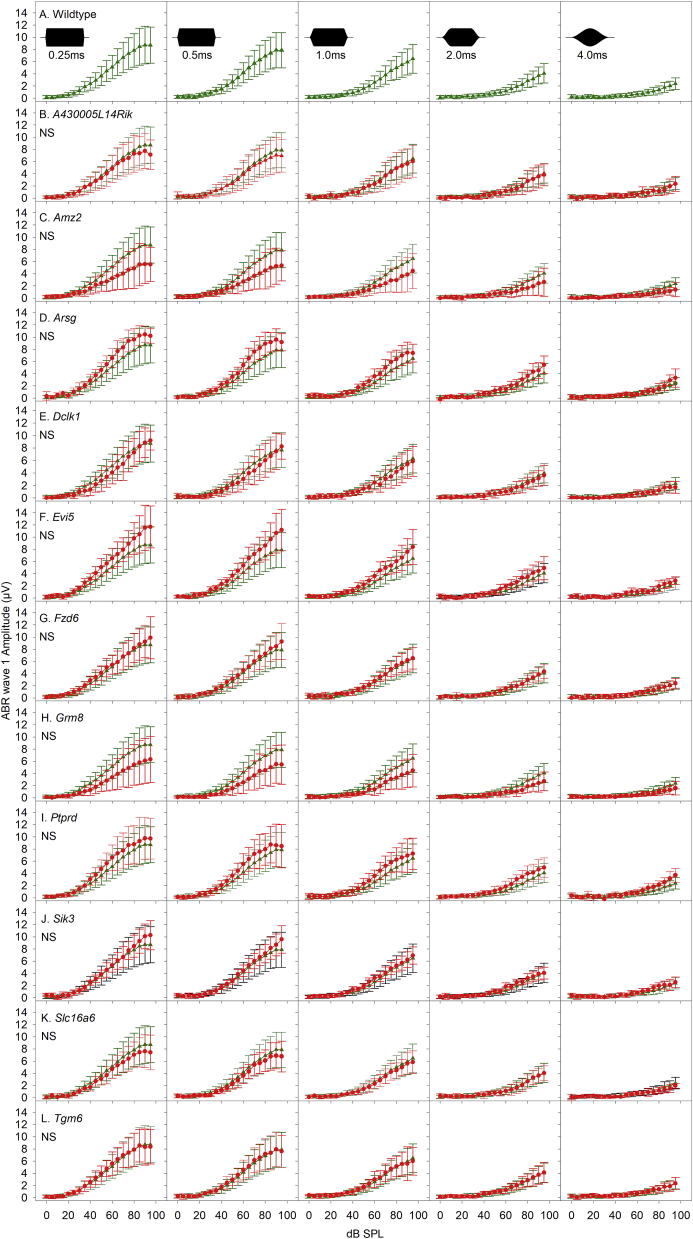
**Responses to changing pure tone ramp time.** Each panel plots mean ABR wave 1 amplitude as a function of stimulus level (dB SPL). Responses to 18 kHz tone pips of 8 m s duration, presented at 42.6/sec, with onset and offset ramp times of 0.25 m s, 0.5 m s, 1.0 m s, 2.0 m s and 4.0 m s are plotted in columns from left-right across the figure. The stimuli are illustrated schematically in the inset panels of the row A. Row A plots mean (+/− SD) wave 1 amplitude from wildtype mice in green (triangles). Rows B-L replot this wildtype data and also plot equivalent data recorded from each mutant line in red (circles). (NS) indicates there is no statistically significant difference between the curves plotted (see also Table 4). (For interpretation of the references to color in this figure legend, the reader is referred to the Web version of this article.)

Thirdly, using 18 kHz stimuli presented 42.6/s, we varied the onset and offset ramp time of an 8 m s tone pip from 0.25, 0.5, 1.0, 2.0–4.0 m, to desynchronise neural firing in the auditory nerve. Each stimulus was presented from 0 to 95 dB SPL in 5 dB increments to generate ABR wave 1 amplitude growth curves (Fig. 8). There were no significant differences in the distribution of wave 1 amplitude at any of the stimulus ramp time functions between the wildtype group or any of the mutant groups (Table 4).

Thus, we found no statistically significant differences in any of the mutant lines tested for any of the threshold and supra-threshold features examined here.

### 3.6 Effects of noise-exposure on wildtype and mutant mice

In two mutant lines (*A430005L14Rik* & *Grm8*) we carried out a further challenge to their auditory system using a noise exposure protocol intended to induce a temporary threshold shift in the mice. We selected *Grm8* because it encodes a metabotropic glutamate receptor subunit, and glutamate excitotoxicity is a potential mechanism to explain synaptic damage triggered by noise-exposure (Puel et al., 1998). Furthermore, a closely related gene (*Grm7*) has been reported to be associated with age-related hearing loss (Friedman et al., 2009). *A430005L14Rik* was chosen because it shows prominent expression in the spiral ganglion (Fig. 2).

**Fig. 9:**
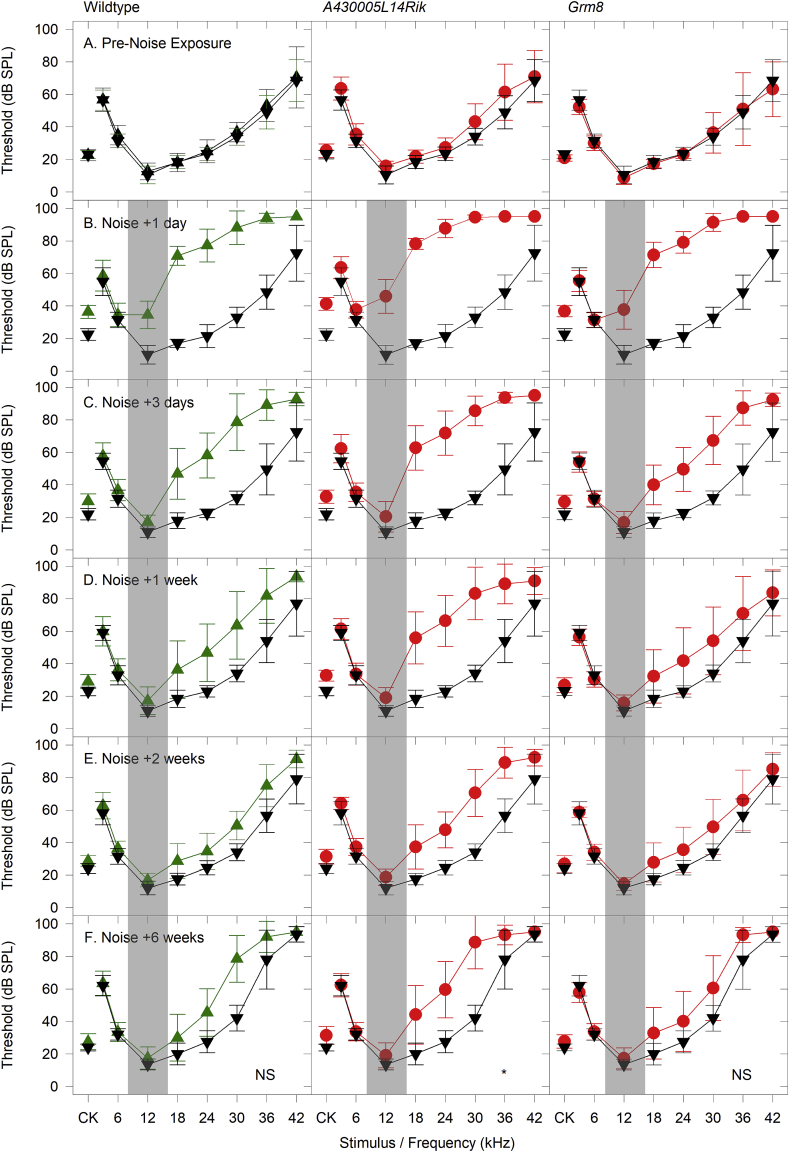
**Threshold sensitivity in Noise-Exposed Mice.** Each panel plots mean (+/− SD) ABR threshold for click stimuli and tone pips ranging from 3 to 42 kHz. Sham control mice (not-exposed to noise, black triangles) are plotted on each panel for comparison with either wildtype noise-exposed mice (green triangles), *A430005L14Rik* homozygous mutant noise-exposed mice (middle column, red circles) or *Grm8* homozygous mutant noise-exposed mice (right column, red circles). Results shown in row A illustrate thresholds in 8 week old mice before the noise-exposure (or sham exposure). Rows B–F show results obtained from the same animals at time points 1 day, 3 days, 1 week, 2 weeks and 6 weeks after the noise-exposure. The 6 week post-exposure time point represents the 14 week old mice subject to the full battery of physiological testing. The grey bar shown in the post-noise exposure panels represents the bandwidth of the noise used (8–16 kHz). Statistical comparisons were performed for the results in row F at 6 weeks after noise exposure (see Table 4). (NS) indicates no significant differences in the threshold curves of 14 week old mice. (*) indicates a significant difference in thresholds from *A430005L14Rik* homozygous mutants compared with sham-control mice (see Table 4 for further detail). (For interpretation of the references to color in this figure legend, the reader is referred to the Web version of this article.)

Mice at 8 weeks old were exposed for 1 h to an octave band of noise (8–16 kHz) presented at 100 dB SPL. ABR thresholds were recorded shortly before exposure and at intervals following the noise exposure. At 14 weeks old we performed the same battery of threshold and supra-threshold ABR testing described above. One day after noise-exposure, thresholds for stimuli within the noise band were mildly elevated and thresholds to stimuli at frequencies above the noise band were severely affected (Fig. 9A and B). Three days after noise-exposure, thresholds for all stimuli had partially recovered (Fig. 9C). This recovery process continued to 6 weeks after noise-exposure (Fig. 9D, E, F), but thresholds never completely recovered at high frequencies although thresholds of wildtype and *Grm8* mutant noise-exposed mice were not significantly different from each other or from sham control mice. *A430005L14Rik* homozygous mutants did show significantly elevated ABR thresholds compared to sham control mice but were not significantly elevated above noise-exposed wildtype mice (Table 4 & Fig. 9F).

**Fig. 10:**
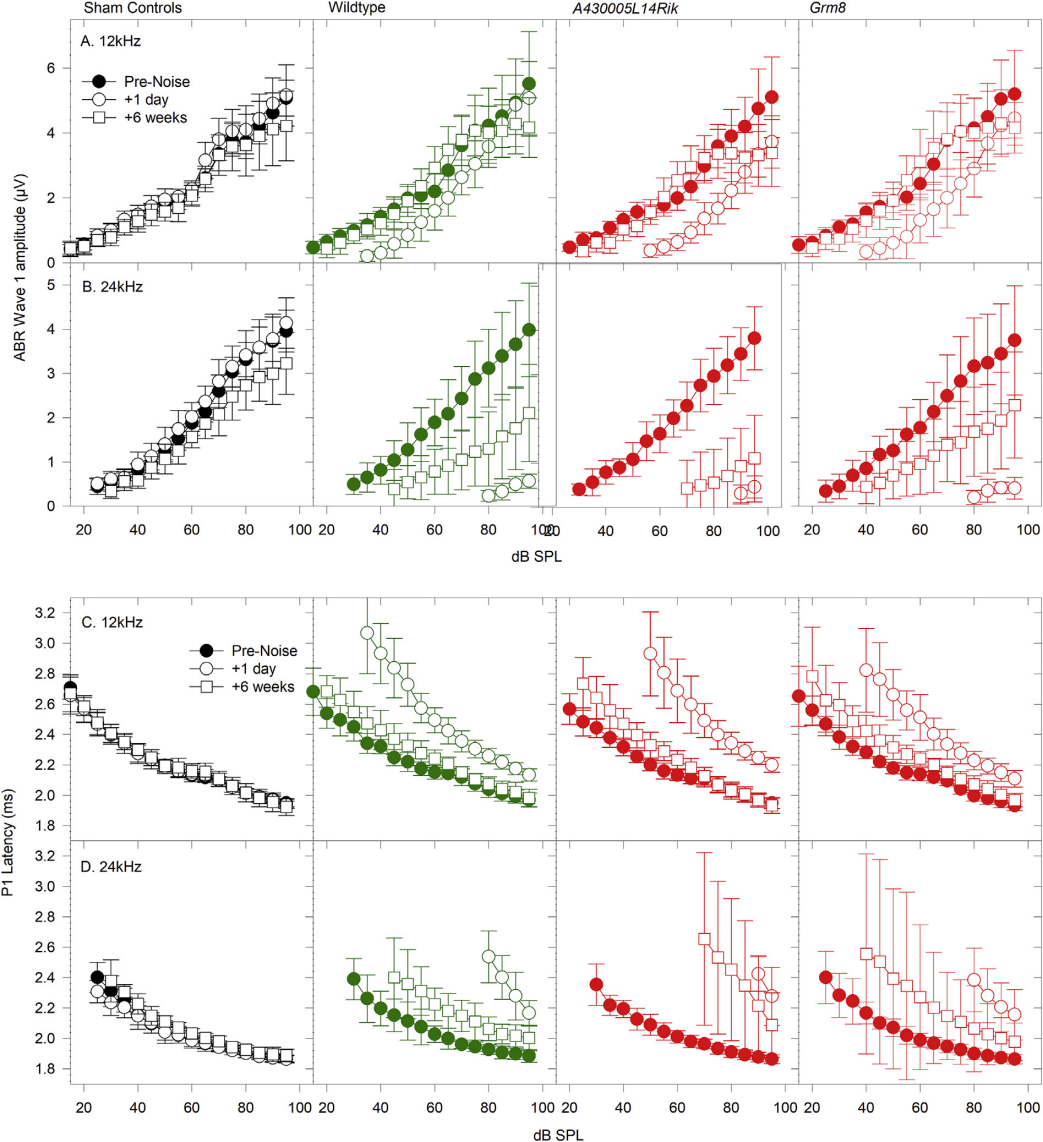
**ABR Wave 1 Amplitude and Latency in Noise-Exposed Mice.** Each panel plots input-output functions for ABR wave 1 amplitude (A,B) or P1 Latency (C,D) as a function of stimulus level (dB SPL), in mice before noise-exposure (filled circles), 1 day after noise-exposure (open circles) and 6 weeks after noise-exposure (open squares). Results obtained using 12 kHz (A,C) and 24 kHz (B,D) tone-pip stimuli are illustrated. Results from sham-exposed control mice are plotted in black; from wildtype noise-exposed mice in green; from *A430005L14Rik* homozygous noise-exposed mice in the third column in red; and from *Grm8* homozygous noise-exposed mice in the fourth column in red. (For interpretation of the references to color in this figure legend, the reader is referred to the Web version of this article.)

We also measured the peak-peak amplitude of wave 1 and the latency of the first positive wave (P1) for ABRs evoked by 12 kHz and 24 kHz tones (Fig. 10). For 12 kHz-evoked ABRs in noise-exposed mice, at 1 day after noise-exposure there was reduction in wave 1 amplitude and an increase in P1 latency, which then recovered back to pre-exposure values at later time points following the exposure. For 24 kHz-evoked ABRs these shifts were much more pronounced and the responses did not fully recover; at 6 weeks after the noise-exposure, wave 1 amplitude remained reduced and P1 latency still showed a rightward shift.

**Fig. 11:**
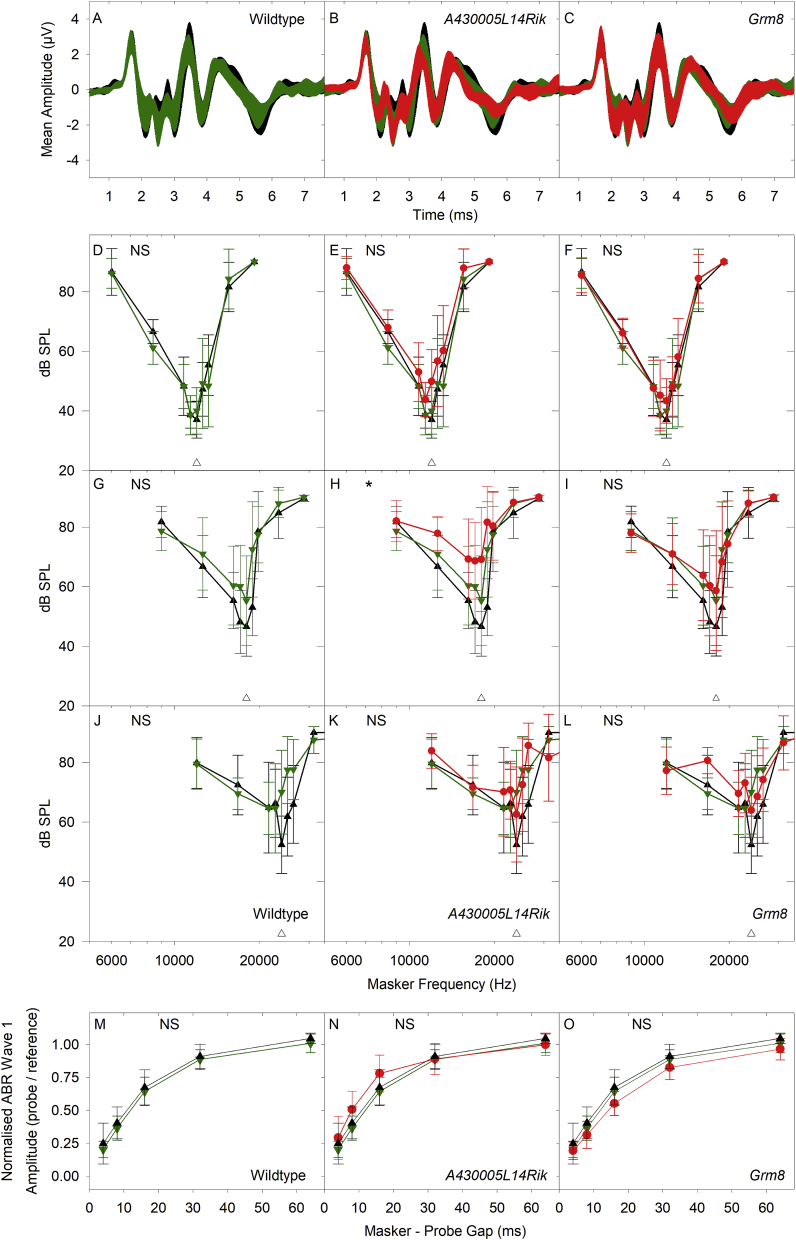
**Click-Evoked ABR waveforms, Forward Masking and Frequency Tuning in mice 6 weeks after Noise-Exposure.** A-C. Mean waveforms (+/− SD) for click-evoked ABRs recorded at 50 dB SL for sham (unexposed) control mice (black; A-C), wildtype noise-exposed mice (green; A) and for noise-exposed *A430005L14Rik* homozygous mutants (red; B) and *Grm8* homozygous mutants (red; C). D-L. Mean frequency tuning curves (see Fig. 5 for detail), using probe tones of 12 kHz (D–F), 18 kHz (G–I) and 24 kHz (J–L), for sham (unexposed) control mice (black triangles; D-L), wildtype noise-exposed mice (green triangles; D-L) and for noise-exposed *A430005L14Rik* homozygous mutants (red circles; E, H, K) and *Grm8* homozygous mutants (red circles; F, I, L). The open triangles close to the frequency axes in J-L indicate the frequency position of the 12 kHz, 18 kHz and 24 kHz probe tones used. (NS) indicates no significant differences in the curves plotted. (*) indicates a significant difference in masked thresholds from *A430005L14Rik* homozygous mutants compared with sham-control mice (see Table 4 for further detail). M-O. Mean forward masking functions (recorded at 50 dB SL, see Fig. 6 for detail) for sham (unexposed) control mice (black triangles; M-O), wildtype noise-exposed mice (green triangles; M-O) and for noise-exposed *A430005L14Rik* homozygous mutants (red circles; N) and *Grm8* homozygous mutants (red circles; O). (NS) indicates no significant differences in the curves plotted. (For interpretation of the references to color in this figure legend, the reader is referred to the Web version of this article.)

At 14 weeks old, waveforms were similar in all groups (Fig. 11A–C). Frequency tuning curves were mostly normal (Fig. 11 D-G, I-L; Table 4), except for the 18 kHz probe tone in *A430005L14Rik* homozygotes which showed significantly higher masked thresholds (Fig. 11H & Table 4).

Forward masking curves showed no significant differences between any of the groups at either stimulus level (Fig. 11M-O; Table 4). The forward masking curves from each group showed excellent fit to an exponential growth to maximum function with R^2^ values ranging from 0.993 to 0.999 (mean ± SD = 0.997 ± 0.003). The maximal adaptation of wave 1 amplitude predicted by these fits (y_0_) ranged from −0.042 to 0.021 with a mean (+/− SD) of −0.012 ± 0.027. The time constant of recovery (tau) of wave 1 amplitude predicted by these fits ranged from 10.5 m s to 20.0 m s with a mean (+/− SD) of 15.8 m s ± 3.9 m s. Maximal recovery (a), predicted from the curve fits ranged from 1.00 to 1.07 with a mean (+/− SD) of 1.04 ± 0.03. Thus, the forward masking curves in the noise-exposed mutants were similar to those of the mutants and controls that were not exposed to noise, as described in section 3.5 and Fig. 6.

**Fig. 12:**
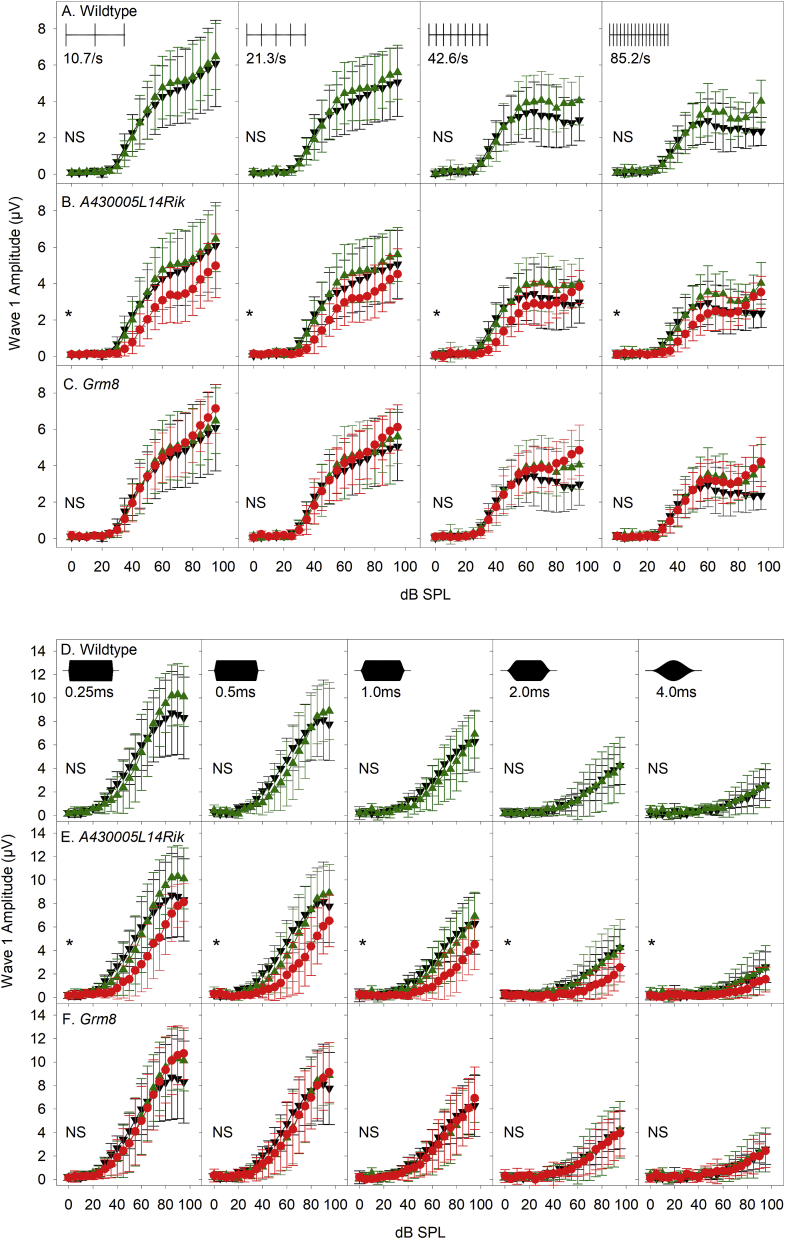
**Responses to changes in click-presentation rate and to changes in tone pip ramp time of mice 6 weeks after noise exposure.** These responses were recorded in 14 week old mice, 6 weeks after noise-exposure when the mice were 8 weeks old. A-C. ABR wave 1 growth functions (vs dB SPL) (see Fig. 7 for further detail), using click presentation rates of 10.65/s, 21.3/s, 42.6/s & 85.2/s (from left to right) for sham (unexposed) control mice (black triangles, A-C), wildtype noise-exposed mice (green triangles, A-C) and for noise-exposed *A430005L14Rik* homozygous mutants (red circles, B) and *Grm8* homozygous mutants (red circles, C). (NS) indicates no significant differences in the curves plotted. (*) indicates a significant reduction in ABR wave 1 amplitude in *A430005L14Rik* homozygous mutants compared with wildtype-exposure or sham-control mice (see Table 4 for further detail). D-F. ABR wave 1 growth functions (vs dB SPL) (see Fig. 8 for further detail), using onset and offset ramp times of 0.25 m s, 0.5 m s, 1.0 m s, 2.0 m s & 4.0 m s (from left to right) for sham (unexposed) control mice (black triangles, D-F), wildtype noise-exposed mice (green triangles, D-F) and for noise-exposed *A430005L14Rik* homozygous mutants (red circles, E) and *Grm8* homozygous mutants (red circles, F). (NS) indicates no significant differences in the curves plotted. (*) indicates a significant reduction in ABR wave 1 amplitude in *A430005L14Rik* homozygous mutants (see Table 4 for further detail). (For interpretation of the references to color in this figure legend, the reader is referred to the Web version of this article.)

Increasing the presentation rate of clicks produced a reduction in amplitude of ABR wave 1, and there was no significant difference in these functions between sham control, wildtype noise-exposed and *Grm8* homozygotes. However, the noise-exposed *A430005L14Rik* homozygotes showed significantly reduced ABR wave 1 amplitudes, suggesting they may have impaired recovery of neural firing compared to wildtype noise-exposed mice (Fig. 12A–C; Table 4).

Increasing the ramp time of a tone stimulus produced a significantly reduced degree of ABR wave 1 growth with stimulus level in noise-exposed *A430005L14Rik* homozygous mutants compared to sham control and wildtype noise-exposed mice (Fig. 12D–F; Table 4). These differences were not seen in noise-exposed *Grm8* homozygotes, or in wildtype noise-exposed mice compared to results from sham control mice. This suggests that noise-exposed *A430005L14Rik* homozygous mutants are more prone to desynchronization of auditory nerve firing compared to control groups.

In summary, the *A430005L14Rik* homozygotes showed slightly impaired recovery from noise exposure measured by thresholds, frequency tuning, response to increased stimulus repetition rates and increased ramp time, but *Grm8* mutants showed no significant differences in recovery compared with wildtype mice.

### 3.7 Progressive hearing loss in *Dclk1* mutant mice

**Fig. 13:**
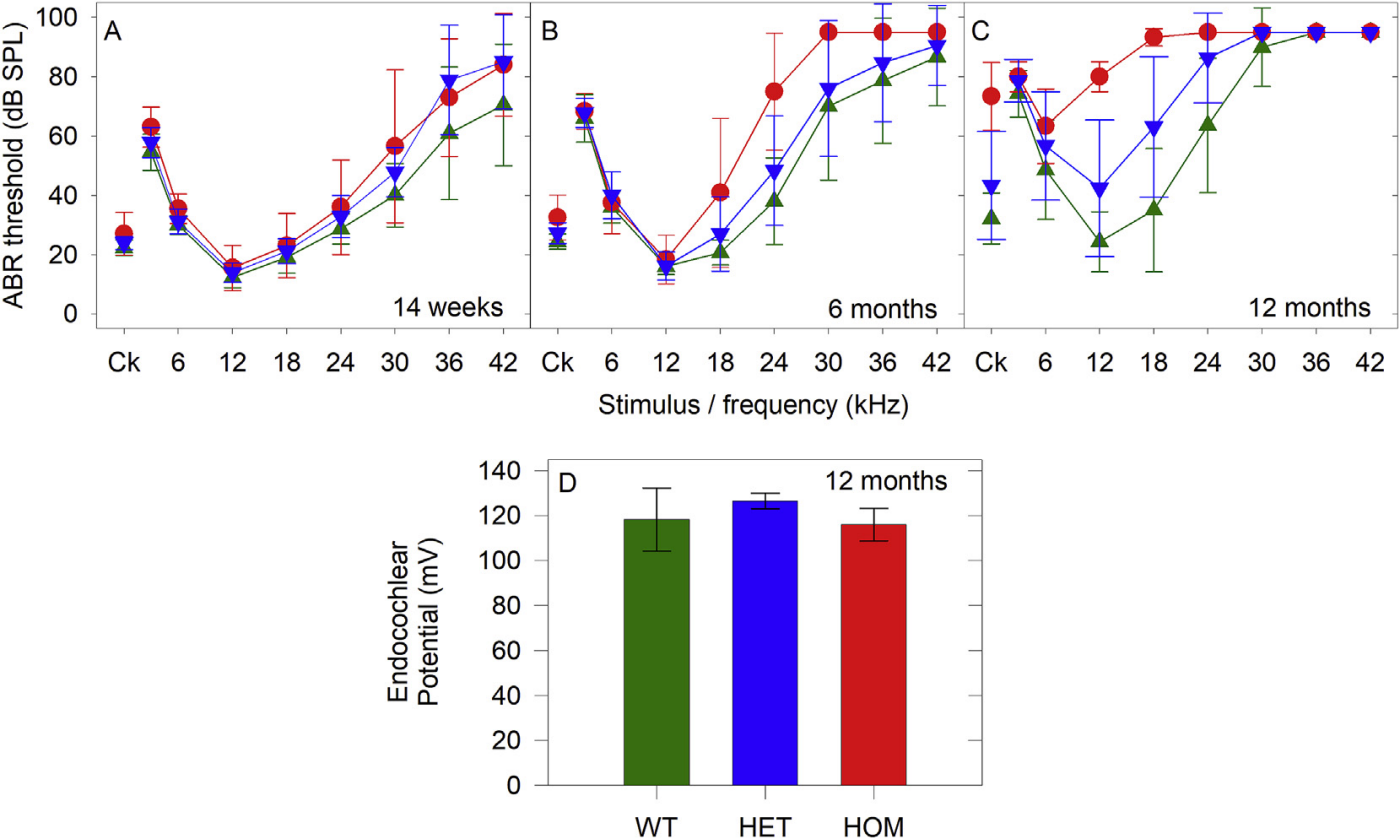
**Dclk1 mutant mice demonstrate a progressive hearing impairment. A-C.** Mean (+/− SD) ABR thresholds for wildtype (green triangles), heterozygote (blue triangles) and homozygote (red circles) *Dclk1* mice are plotted for mice aged 14 weeks (A. wildtype, n = 11, 14.22 ± 0.95wk; het, n = 9, 14.30 ± 1.03wk; hom, n = 10, 14.16 ± 0.34wk), 6 months (B. wildtype, n = 15, 26.15 ± 0.46wk; het, n = 18; 26.08 ± 0.31wk; hom, n = 6, 25.81 ± 0.46wk) and 12 months (C. wildtype, n = 7, 53.43 ± 1.73wk; het, n = 15, 53.70 ± 1.53wk; hom, n = 3, 54.52 ± 0.41wk). D. Mean (+/− SD) endocochlear potential is plotted for 12 month old wildtype (WT, n = 4, 54.5 ± 3.4wk green), heterozygote (HET, n = 2, 51.6 ± 0.6wk, blue) and homozygote (HOM, n = 2, 57.4 ± 0.4wk, red) *Dclk1* mice. (For interpretation of the references to color in this figure legend, the reader is referred to the Web version of this article.)

There was a small (but not significant) threshold elevation in some individual homozygous *Dclk1* mutants for frequencies of 24 kHz and greater at 14 weeks old (Dunn’s Method of multiple comparisons, p > 0.121). For this reason, these mice were followed up to 12 months old (Fig. 13A–C). By 6 months of age, thresholds in all mice tested showed high frequency threshold elevations. There was no significant difference in thresholds between wildtype and heterozygote mice (Dunn’s Method of multiple comparisons, p = 0.465), but thresholds for the *Dclk1* homozygous mutants were significantly elevated above wildtypes (Dunn’s Method of multiple comparisons, p = 0.015). A similar pattern was observed at 12 months old, in that there was no significant difference between thresholds of wildtype and heterozygote mice (Dunn’s Method of multiple comparisons, p = 0.069), however thresholds for the *Dclk1* homozygous mutants were significantly elevated (Dunn’s Method of multiple comparisons, p = 0.003). As *Dclk1* was strongly expressed in the marginal cells of the stria vascularis, we measured endocochlear potentials to assess strial function. However, at 12 months old, endocochlear potentials were normal (100 mV or more) in all three genotypes (Fig. 13D, one-way ANOVA F = 0.495, df = 2, p = 0.637).

## 4 Discussion

### 4.1 *Dclk1* is involved in age-related progressive hearing loss

Of the 17 genes that were promising candidates from GWAS of human age-related hearing loss when we started this study, one was found to be involved in hearing loss in the mouse, doublecortin-like 1 (*Dclk1*). The DCLK1 protein is highly conserved: 98% of the human DCLK1 protein sequence matches the mouse Dclk1 protein sequence. Data from gnomAD (v2.1.1) suggests that *DCLK1* is highly intolerant to loss of function (LoF); only four variants likely to cause loss of function have been reported and all are rare. The expected number of LoF variants, based on a depth-corrected probability of mutation, is 40, resulting in a pLI (probability of being loss of function intolerant) of 1. The mouse *Dclk1* mutation knocked down mRNA expression of the gene (Fig. 1) which was associated with a progressive increase in ABR thresholds from 14 weeks to 12 months old (Fig. 13), with heterozygotes showing an intermediate phenotype. As *Dclk1* has prominent expression in marginal cells of the stria vascularis (Fig. 2; see also Girotto et al., 2014), we measured endocochlear potentials (EP) to assess strial function. However, EP magnitudes were normal in homozygous *Dclk1* mutants, heterozygotes and wildtypes at 12 months old, suggesting that the cause of the hearing loss is not due to the dysfunction of the stria vascularis. Apart from the GWAS reports leading to the current study (Girotto et al., 2011, 2014), *Dclk1* has no known role in hearing, although it has been widely studied in other systems. For example, it has roles in colorectal, liver and pancreatic cancers (eg. Fan et al., 2017; Fesler et al., 2017; Kawamura et al., 2017) and in dendritic remodelling and synapse maturation (Shin et al., 2013). The related gene doublecortin (*Dcx*) has well described roles in neurogenesis and neuronal migration in the rat dorsal cochlear nucleus (Manohar et al., 2012; Paolone et al., 2014). The gene *DCDC2* (Doublecortin domain containing 2a, *Dcdc2a*) underlies human recessive deafness DFNB66 (Grati et al., 2015). However, there is no apparent functional link between *Dclk1* (a serine/threonine protein kinase) and either *Dcx* or *Dcdc2a*, other than that they all contain doublecortin domains, which are thought to interact with microtubules to stabilise them (Burger et al., 2016). As marginal cells of the stria are rich in microtubules, Dclk1 might contribute to their stabilisation even though it is not essential for strial function.

### 4.2 *A430005L14Rik* is involved in susceptibility to noise damage

Following noise exposure wildtype mice showed raised thresholds, reduced amplitudes and prolonged latencies of wave 1, which all recovered partly over the following two weeks. However, the levels never returned completely to the levels that were recorded before noise exposure or in sham-exposed littermates, even by 6 weeks after exposure (Fig. 9, Fig. 10), although this was significant only for the *A430005L14Rik* mutants (Table 4). These noise-exposed mutants also showed raised and broader tuning curves at 18 kHz suggesting poorer frequency discrimination and impaired temporal processing properties shown by reduced ABR wave 1 amplitudes with increasing stimulus repetition rate and longer ramp time of the stimulus tonebursts (Fig. 11, Fig. 12). Whilst there is little known about the *A430005L14Rik* gene, it has been reported to be expressed in the nucleus (Stadler et al., 2012). We have shown here that it is strongly expressed in the spiral ganglion (Fig. 2), suggesting a primary neural role in the pathology following noise exposure. The orthologous human protein, C1orf174, shows 64% identity in sequence to the mouse A430005L14Rik protein sequence, and data from gnomAD (v2.1.1) suggests that the *C1orf174* gene is relatively tolerant of variation, with a pLI of 0.

The *Grm8* gene expresses a subunit of metabotropic glutamate receptors (mGluR8) which belongs to a family of receptors thought to decrease NMDA receptor activity and risk of excitotoxicity (Ambrosini et al., 1995). Thus, knockout of *Grm8* expression might have increased the damaging effects of noise-exposure in the cochlea via excitotoxicity, resulting in abnormal auditory function. However, *Grm8* mutant mice did not show any significant impairment of auditory function compared to wildtype noise-exposed mice or sham controls. It may be that there is functional redundancy in this gene family and that expression of a related receptor subunit compensated for altered expression of *Grm8*.

### 4.3 Role of single-gene mutations in ARHL

Fifteen of the 17 genes investigated here (Table 1, Table 2) showed no significant impact on auditory phenotypes in mutant mice aged 14 weeks old, despite the use of a battery of physiological tests designed to uncover suprathreshold functional deficits in cochlear and auditory nerve function. These genes were chosen on the basis of suggestive involvement in hearing from GWAS in humans and their expression in the cochlea (Girotto et al., 2011, 2014; Wolber et al., 2014), and were the best available candidates at the time we started this study (Table 1, Table 2).

Since that time, three of the genes investigated here have been further implicated in hearing loss. Firstly, Krinner et al. (2017) suggested that deficiency of Rimbp2 protein results in altered synaptic vesicle release from inner hair cells, causing a longer first spike latency in auditory nerve discharge patterns, reduced amplitude of ABR wave 1 and mild elevations of ABR thresholds. However, we see normal auditory sensitivity in the *Rimbp2*^*em1(IMPC)Wtsi*^ mice studied here, with normal waveform shape. The difference in wave 1 amplitude might be explained by the level chosen to compare; we compared waveforms at a set level above threshold for each mouse, dB SL, while Krinner and colleagues used a set dB SPL to compare. The difference in threshold is more difficult to explain but may relate to a different allele or different genetic background of the two mutants studied. The allele we analysed was generated by CRISPR targeted deletion of exon 7 (of 22) of transcript Rimbp2-201 (ENSMUST00000111346.5), compared to a loxP mediated excision of exon 17 (encoding part of the second SH3 domain of the Rimbp2 protein; Grauel et al., 2016).

Secondly, Choucair et al. (2015) have implicated the *PTPRD* gene in human deafness, reporting a child with a homozygous *PTPRD* intragenic deletion leading to a 40 dB audiometric hearing loss along with trigonocephaly and intellectual disability. Whilst this supports the GWAS findings of Girotto et al. (2014), the mouse mutant investigated in the current study, carrying the *Ptprd*^*tm2a(KOMP)Wtsi*^ targeted allele, does not show a significant degree of hearing impairment or auditory dysfunction. The reason for the difference may be that the mouse allele we analysed was leaky, with about 12% of the normal level of transcript detected, while the proband affected by the human mutation carried two null alleles.

The third gene from our list associated with deafness is *ARSG*, involved in late-onset atypical Usher syndrome, with hearing and vision deficits appearing around the age of 40 (Khateb et al., 2018). Five patients were all found to carry a homozygous missense variant, c.133G > T, causing an aspartate to tyrosine amino acid change (pD45Y) located in the highly-conserved catalytic site and confirmed to eliminate functional activity of the encoded enzyme arylsulfatase G. This enzyme has been characterised as a lysosomal sulfatase (Frese et al., 2008) but did not lead to the predicted glycosaminoglycan excretion in patients (Khateb et al., 2018). As the onset of hearing loss is relatively late in the patients, we may not have found raised thresholds in the mouse *Arsg* mutant because we only tested hearing in this line up to 14 weeks old.

There are several possible explanations for the scarcity of auditory phenotypes in these 15 mutant lines. Firstly, the lack of raised thresholds could be due to incomplete knockdown of targeted gene expression in the tm1a allele (Fig. 1). However, our previous experience in using the tm1a allele of other genes indicates that even when there is limited knockdown of the mRNA level, there is often a detectable phenotype (e.g. White et al., 2013). Most of the mutant alleles we studied showed other phenotypes, suggesting that the knockdown was effective in other tissues (Table 2). Secondly, the majority of lines were tested at 14 weeks old only and we may have detected evidence of accelerated hearing loss if we had looked at older mice. This was found to be the case with the *Dclk1* mutant mice where hearing loss was only detected after 14 weeks. Nonetheless, we have detected multiple lines with raised ABR thresholds and/or abnormal waveforms by screening at 14 weeks old in a larger study (Ingham et al., 2019) and we selected 14 weeks old as an age when hearing is fully mature but before the onset of progressive hearing loss commonly observed in the C57BL/6N genetic background. Thirdly, any effects on hearing of the loci detected in the GWAS may be mediated by expression of a modified protein with abnormal function (gain of function), rather than by a general reduction of expression level as in the mouse mutants studied. However, as so many of the significant GWAS loci are intergenic, it is generally thought that the impact on phenotype is most often due to effects of variants on expression levels. Fourthly, GWAS are based on genomic markers that are not usually the causative variant but instead mark a region that is closely linked to a causative variant. The genes we selected were the closest to the most significant peaks of marker linkage in each GWAS, but the nearest gene may not be the one underlying the phenotype. For example, we have previously reported a mutation which led to deafness by a long-range *cis* effect on a nearby gene: the *Slc25a21*^*tm1a(KOMP)Wtsi*^ targeted mutation causes raised ABR thresholds by reducing expression of the nearby gene *Pax9* (Maguire et al., 2014). Also, the peaks in the GWAS could represent regulatory regions that are far from their target gene, and cases of regulatory sequences up to 1 Mb away from the gene have been reported. Thus, the closest gene may not be the gene associated with hearing loss.

The fifth possible explanation is that mutations of these genes may lead to enhanced sensitivity to environmental damage and we tested only two mutant lines after noise exposure. Some of the other mutant lines may have shown an exaggerated response to an environmental challenge if we had tested them. Likewise, we only tested these mutant alleles on a single inbred genetic background, C57BL/6N, and it may be that if we had used a different background or a mixture of backgrounds then we may have detected an effect. We decided to use an inbred background to minimise the biological variation that would be introduced by a mixed background, thus minimising the experimental noise in our measurements. The C57BL/6N background carries loci known to predispose to hearing loss, including the single base variant of the *Cdh23* gene, *Cdh23*^*753G>A*^, which may interact with the new mutant alleles to exacerbate any effect on hearing. However, there are many other genetic variants in the genetic background of mice and humans, known and unknown, that may interact with the new mutant alleles we studied here, and any one of these may tip the balance from normal maintenance of hearing to hearing loss in an individual.

A further possibility is that the genes investigated in the current study may be involved in hearing but make such a small contribution to the phenotype that it cannot be detected even when a mutation with a severe effect is analysed as in our study. This would not be surprising given that many loci identified by GWAS of other diseases appear to have a very small effect size. The GWAS candidate genes we studied may have been false positive findings. This would not be too surprising because although they were the best candidates at the onset of this study, these genes have not been replicated since the original publications and they were reported as having “suggestive” statistical significance at the time after correction for multiple testing.

These findings emphasise the difficulty in understanding the genetic architecture of common, heterogeneous diseases with adult onset like ARHL (eg Tam et al., 2019). The design of GWAS depends on the assumption that ancient mutations affecting the phenotype are still closely linked to the markers used and are widely spread throughout the population under study. GWAS will not pick up more recent mutations that lead to hearing loss because these will not have had time to spread through the population so will be too rare to detect by GWAS. Nonetheless, the very recent reports from the UK Biobank dataset of significant genome-wide associations of a number of genes, including some candidates that were already known to be involved with childhood deafness, may lead to an improved yield of genes involved in ARHL (Wells et al., 2019; Kalra et al., 2019).

An alternative view is that finding two genes involved in aspects of hearing impairment out of 17 studied is probably a reasonable hit rate given the nature of GWAS. A recent meta-analysis of published Genome Wide Association Studies suggested that significant GWAS hits may explain such a small fraction of the total genetic variance in the particular disease studied that such hits may be general omnigenic “fitness indicators” rather than clues to disease-specific molecular pathways (Boyle et al., 2017). Our findings in this set of candidate genes for hearing loss fits this hypothesis and suggests that the genetic architecture of age-related hearing is still unclear.

## CRediT authorship contribution statement

**Neil J. Ingham:** Conceptualization, Methodology, Software, Validation, Formal analysis, Investigation, Data curation, Writing - original draft, Writing - review & editing, Visualization, Supervision, Project administration, Funding acquisition. **Victoria Rook:** Investigation, Visualization. **Francesca Di Domenico:** Investigation. **Elysia James:** Investigation. **Morag A. Lewis:** Methodology, Software, Validation, Data curation, Writing - review & editing. **Giorgia Girotto:** Conceptualization. **Annalisa Buniello:** Conceptualization, Methodology, Validation, Investigation, Data curation, Writing - review & editing, Visualization, Supervision, Project administration, Funding acquisition. **Karen P. Steel:** Conceptualization, Resources, Writing - review & editing, Supervision, Project administration, Funding acquisition.
